# A Complementary Dual‐Mode Ion‐Electron Conductive Hydrogel Enables Sustained Conductivity for Prolonged Electroencephalogram Recording

**DOI:** 10.1002/advs.202405273

**Published:** 2024-08-08

**Authors:** Hengjie Su, Linna Mao, Xiaoqi Chen, Peishuai Liu, Jiangbo Pu, Zhuo Mao, Tomoko Fujiwara, Yue Ma, Xinyang Mao, Ting Li

**Affiliations:** ^1^ Institute of Biomedical Engineering Chinese Academy of Medical Sciences & Peking Union Medical College Tianjin 300192 China; ^2^ Department of Biomedical Engineering Tiangong University Tianjin 300187 China; ^3^ Department of Chemistry The University of Memphis Memphis TN 38152 USA; ^4^ Department of Biomedical Engineering Tianjin Medical University Tianjin 301700 China

**Keywords:** conductive hydrogel, dual‐mode conducting mechanism, electroencephalogram (EEG), graphite nanoparticles, hyaluronic acid

## Abstract

Conductive gel interface materials are widely employed as reliable agents for electroencephalogram (EEG) recording. However, prolonged EEG recording poses challenges in maintaining stable and efficient capture due to inevitable evaporation in hydrogels, which restricts sustained high conductivity. This study introduces a novel ion‐electron dual‐mode conductive hydrogel synthesized through a cost‐effective and streamlined process. By embedding graphite nanoparticles into ionic hyaluronic acid (HAGN), the hydrogel maintains higher conductivity for over 72 h, outperforming commercial gels. Additionally, it exhibits superior low skin contact impedance, considerable electrochemical capability, and excellent tensile and adhesion performance in both dry and wet conditions. The biocompatibility of the HAGN hydrogel, verified through in vitro cell viability assays and in vivo skin irritation tests, underscores its suitability for prolonged skin contact without eliciting adverse reactions. Furthermore, in vivo EEG tests confirm the HAGN hydrogel's capability to provide high‐fidelity signal acquisition across multiple EEG protocols. The HAGN hydrogel proves to be an effective interface for prolonged high‐quality EEG recording, facilitating high‐performance capture and classification of evoked potentials, thereby providing a reliable conductive medium for EEG‐based systems.

## Graphical abstract

A complementary ion‐electron dual‐mode conductive hydrogel, prepared through a straightforward and simple method, exhibits prolonged high conductivity and superior biocompatibility, making it a reliable agent for long‐term electroencephalogram (EEG) recording. The ionic hyaluronic acid embedded with graphite nanoparticles (HAGN) hydrogel maintains higher conductivity for over 72 h and ensures high‐fidelity signal acquisition for various EEG protocols, including steady‐state visually evoked potential (SSVEP) and P300.

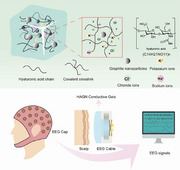

## 1 Introduction

Long‐term electroencephalogram monitoring is essential for various medical applications, including epilepsy monitoring,^[^ ^1^, ^2^, ^3^, ^4^ ^]^ intensive care unit,^[^ ^5^ ^]^ intraoperative EEG monitoring,^[^ ^6^ ^]^ operating room,^[^ ^7^, ^8^ ^]^ and emergency department.^[^ ^9^ ^]^ Additionally, prolonged EEG recording also plays a significant role in evaluating sleep disorders,^[^ ^10^, ^11^ ^]^ psychiatric conditions, and movement disorders,^[^ ^12^ ^]^ highlighting its importance in medical research and clinical practice.^[^ ^13^, ^14^, ^15^, ^16^ ^]^ Conventional EEG signal acquisition setups rely on wet silver/silver chloride (Ag/AgCl) electrodes combined with electrolytic hydrogels, which are regarded as the gold standard in non‐invasive EEG monitoring.^[^ ^15^, ^17^, ^18^, ^19^ ^]^ Conductive hydrogel has emerged as a promising alternative for EEG signal acquisition in recent years.^[^ ^20^, ^21^, ^22^ ^]^ To meet the requirements for long‐term EEG monitoring, several key factors must be considered in the preparation of gel electrodes. These include strong adhesion to the skin to maintain uninterrupted contact during minor body movements and perspiration,^[^ ^23^ ^]^ high conductivity to ensure superior signal quality and sustained performance. Furthermore, addressing potential allergic reactions from prolonged gel‐skin contact and ensuring biocompatibility is crucial.^[^ ^24^, ^25^ ^]^ In essence, developing conductive gels that integrate prolonged high electrical conductivity, mechanical durability, and superior biocompatibility remains a significant challenge for continuously recording high‐quality EEG signals.

Conductive hydrogels have shown various metrics and gained considerable attention as a domain class of electrode material in recent years.^[^ ^33^, ^34^, ^35^, ^36^ ^]^ A variety of conductive hydrogels have been developed for utilization in EEG signal recording.^[^ ^30^ ^]^ Alginate‐based hydrogel that solidifies rapidly for easier post‐test cleaning has been explored as a substitute for conventional electrolytic gels.^[^ ^21^ ^]^ Hydrogels based on gelatin and polydopamine methacrylamide‐polyacrylamide have been designed for enhanced adhesion.^[^ ^31^, ^32^ ^]^ Ionic hydrogels with inorganic salt ions show high conductivity and are widely used in both academic research and commercial products like Ten20 EEG conductive paste, Greentek GT5 Freeprep conductive gel, and Dermedics EEG hydrogel.^[^ ^26^, ^27^, ^28^, ^29^ ^]^ However, the high conductivity of ionic hydrogels is highly dependent on water content, which inevitably evaporates over time and leads to conductivity instability. Many efforts have been devoted to developing ionic hydrogels that retain water content for extended periods to ensure sustained conductivity, but these often involve complex chemical synthesis and potential toxic hazards.^[^ ^37^ ^]^ Despite numerous research aimed at enhancing the electrical conductivity of ionic gels in recent years, long‐term stability remains a challenge for clinical applications and sustainable EEG signal acquisition. Therefore, addressing the signal deterioration as water evaporation in ionic hydrogels is crucial to endowing prolonged high conductivity in long‐term EEG recording.

In this study, we developed a complementary ion‐electron dual‐mode conductive hydrogel via a straightforward and cost‐effective method by incorporating graphite nanoparticles (GNs) and salt ions into hyaluronic acid (HA) gel. The resulting hyaluronic acid‐graphite nanoparticles (HAGN) ionic hydrogel exhibit sustained high conductivity in both wet and dry states due to the complementary effect of ionic and electronic conducting mechanisms. The electron‐conducting mechanism facilitated by graphite nanoparticles complements the dominant ion‐conducting mechanism in the ionic conductive hydrogel, compensating for the electrical performance degradation caused by water evaporation. The HAGN hydrogel maintained superior conductivity for over 72 h in comparison with commercial gels, and consistently exhibited low skin contact impedance performance in both wet and dry states. Additionally, the innovative utilization of HA as a polymer matrix minimizes the risk of on‐skin contact, ensuring convincing biocompatibility. The introduction of carbon‐based nanomaterials enhanced electrochemical and mechanical properties as well. As an excellent conductive agent combining prolonged conductive stability, comparable electrochemical and mechanical properties, and excellent biocompatibility, HAGN hydrogel has great potential for long‐term EEG acquisition. Subsequently, the HAGN hydrogel conducted in three EEG protocols, demonstrating its suitability for real‐world applications compared to a commercial gel. Consequently, the complementary dual‐mode conducting HAGN hydrogel holds significant potential for capturing and classifying evoked potentials with high performance, providing a long‐term reliable interface for EEG‐based systems.

## 2 Results

### 2.1 Preparation and Characterization of HAGN Hydrogels

**Figure 1:**
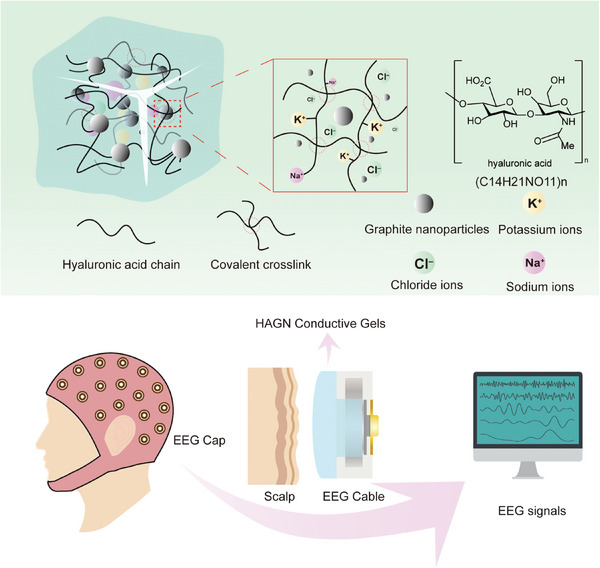
Schematic illustration of the HAGN conductive hydrogels for long‐term EEG signal acquisition.

Ionic conductive hydrogels typically consist of polymer matrices, ionic conductors, and conductive additives. Hyaluronic acid (HA) is a naturally occurring linear polysaccharide, widely used as implanted fillers due to its biocompatibility, biodegradability, and water retention ability.^[^ ^38^ ^]^ A conductive hydrogel was synthesized by incorporating graphite nanoparticles (GNs) and ionic conductors into hyaluronic acid, resulting in hyaluronic acid‐graphite nanoparticles ionic hydrogel (HAGN hydrogel). Graphite nanoparticles are a versatile candidate for polymer composites due to their lightweight, high aspect ratio, substantial electrical conductivity, and biocompatibility.^[^ ^39^ ^]^ The platelet form of GNs facilitates their incorporation into polymeric matrices using solvents, enhancing the overall properties of the composite.^[^ ^40^, ^41^ ^]^ Sodium chloride (NaCl) and potassium chloride (KCl) were added as ionic conductors to enhance electrical conductivity in the flowable gel state, while glycerol was added to maintain essential water retention. GNs were incorporated as conductive additives to ensure conductivity in both wet and dried states. **Figure** **1** shows the schematic diagram of the HAGN hydrogel with a dual‐mode conducting mechanism, which ensures the long‐term conductive stability of the HAGN hydrogel and makes it ideal for continuous EEG signal acquisition. The detailed preparation process of HAGN is shown in Figure S1 (Supporting Information). When the HAGN hydrogel is in a wet state, the conductivity is primarily ensured via ions including Cl^−^, K^+^, and Na^+^. As the HAGN hydrogel gradually dries, it transitions into a graphite‐doped conductive film, and the dominance of ion‐conducting mechanism gradually transitions to the electron‐conducting mechanism, facilitated by the graphite nanoparticles. Once the water content stabilizes, the conductivity determined by the internal distribution of graphite nanoparticles remains stable accordingly, endowing the HAGN conductive gel with sustained high conductivity over time.

**Figure 2:**
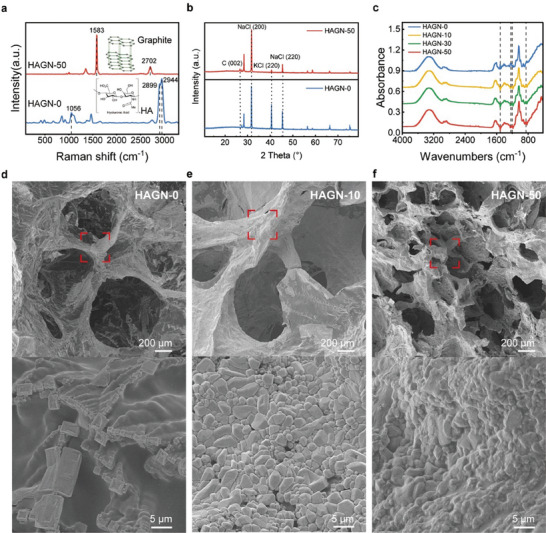
Chemical and morphological characterization of HAGN hydrogels. a) Raman spectra of HAGN with graphite (HAGN‐50)/without graphite (HAGN‐0). b) XRD patterns of HAGN with graphite (HAGN‐50)/without graphite (HAGN‐0). c) FTIR spectra of HAGN with different concentrations of graphite: 0, 10, 30, and 50 mg ml^−1^; SEM images of the HAGN hydrogel with various graphite concentrations: d) HAGN‐0; e) HAGN‐10; f) HAGN‐50.

The Raman spectrum was employed to characterize the chemical component of HAGN hydrogel. **Figure** **2**a compared the Raman spectra of the non‐doped HAGN (HAGN‐0) and HAGN doped with 50 mg ml^−1^ (HAGN‐50). The Raman characteristics peaks of HA were observed at ≈1056 cm^−1^, corresponding to the C─C and C─O stretching, and at ≈2899 and ≈2904 cm^−1^, corresponding to C‐H and N‐H stretching modes.^[^ ^42^ ^]^ While the Raman characteristic peaks of HA are extremely weak,^[^ ^42^, ^43^ ^]^ doping with graphite nanoparticles enhances the Raman spectrum of the HAGN hydrogel, revealing the prominent characteristic graphite peaks at ≈1583 and ≈2702 cm^−1^. Further characterization of the HAGN hydrogels before and after doping with graphite nanoparticles was performed using an X‐ray Diffraction Pattern (XRD).^[^ ^44^, ^45^ ^]^ The characteristic peaks of sodium chloride and potassium chloride crystalline grains were observed in both HAGN‐0 and HAGN‐50 (Figure 2b). After doping with graphite nanoparticles, a characteristic peak corresponding to graphite was observed in the XRD pattern of the HAGN hydrogel at 2*θ* = 26.7° (Figure 2b; Figure S2, Supporting Information). The chemical components of HAGN were further characterized via Fourier‐transform infrared spectroscopy (FTIR). The characteristic peaks of HA hydrogel were clearly observed at ≈1030 and ≈1100 cm^−1^, which correspond to C─O─C, C─O, and C─O─H stretching, and the peaks at ≈1600 and ≈1630 cm^−1^ correspond to the N─H bend and C═O stretching (Figure 2c). The introduction of graphite nanoparticles has a negligible effect on the chemical compositions of the HA ionic gel.

Scanning Electron Microscopy (SEM) was employed to characterize the structural and morphological features of HAGN hydrogels. Figure 2d shows the SEM of HAGN without graphite doped, the obvious 3D structure in HA indicates a higher water content and further ensures the effectiveness of the ion‐conducting mechanism. As shown in the magnifying SEM image in Figure 2d, there are many tetragonal chloride crystalline grains covered on the surface of the HA polymer skeleton. Figure 2e,f shows the SEM images of HAGN hydrogels with graphite‐doped concentrations of 10 and 50 mg ml^−1^ respectively, which indicate that the HAGN hydrogel exhibits an obvious 3D porous structure representing a quite large water content. Figure S3 (Supporting Information) presents SEM images of HAGN hydrogels with graphite concentrations of 0, 10, 20, 30, 40, and 50 mg ml^−1^, revealing a slight effect on the porous structure, all of which ensure the high water content features of the HAGN hydrogels. Moreover, the magnified SEM images demonstrated that the graphite nanoparticles are evenly mingled with the chloride crystalline grains, and the aggregation degree of the graphite particles increases with the increase of graphite concentration accordingly. The size of salt crystals, highly influenced by salt concentration,^[^ ^46^ ^]^ grows more rapidly at higher concentrations, forming larger crystal particles that directly affect the uniformity of graphite particle distribution, which further affects the conductivity of HAGN in a solidified film state (Figure S4, Supporting Information).

### 2.2 Electrical, Electrochemical, and Mechanical Performances of the HAGN

**Figure 3:**
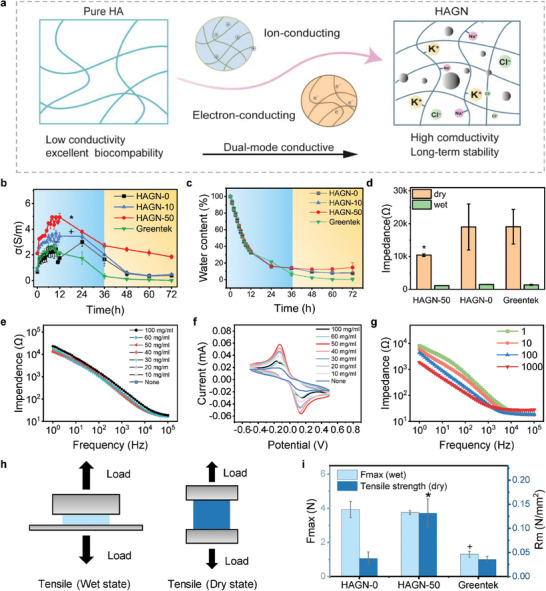
Electric and mechanical properties of HAGN hydrogels. a) Schematic illustration of complementary ion‐electron dual‐mode conducting mechanism. b) The conductivity of the HAGN with/without graphite contained and commercial gel for 72 h. c) Water content curves over time for various HAGN hydrogels (HAGN‐0, HAGN‐10, HAGN‐50) and commercial gel (Greentek gel). d) Skin contact impendence of HAGN with/without graphite contained and commercial gel at 1 kHz, under wet/dry state, respectively. e) EIS curves of the HAGN hydrogel with different graphite concentrations. f) Cyclic voltammetry (CV) curves of the HAGN hydrogels with different graphite concentrations. g) EIS curves of HAGN (6 wt.% NaCl/KCl, 50 mg ml^−1^ graphite) after 1^st^, 10, 100, 1000 cycles of cyclic voltammetry test. h) Setup for tensile test when the HAGN hydrogel is in dry and wet condition. i) Tested tensile strength (Rm) and tested maximum force of HAGN film with a water content of 25%, the Rm is defined as: Rm = F_max_ / S_cross‐sectional area_. Data in (b‐d) i) are presented as mean ± standard deviation (Mean ± SD), with *n* = 3. P values were determined using two‐way analysis of variance (ANOVA) with Tukey's post‐hoc test in b and c, and one‐way ANOVA with Tukey's post‐hoc test in (d) and (i). * and + denote significant differences between groups (*p* < 0.05).

Compared to pure HA gels, the sustained conductivity of HAGN hydrogels is attributed to the complementary ion‐electron dual‐mode conducting mechanism, which combines ionic conductivity facilitated by salt ions and electronic conductivity facilitated by graphite nanoparticles. The complementary mechanism synergistically guarantees long‐term conductivity in both wet and dry state (**Figure** **3**a). The electrical conductivity (σ) and water content variation curves for HAGN hydrogels with different GNs concentrations and commercial gel over 72 h were tested and are shown in Figure 3b,c, respectively. The commercial gel (Greentek) showed an obvious downward trend in conductivity after 24 h, reaching only 0.33 S m^−1^ at 36 h, and further declining to 0.0078 S m^−1^ at 72 h. In contrast, the HAGN hydrogels all displayed a similar trend but higher conductivity levels throughout the testing period. In the wet state, the conductivity is primarily driven by the dominant ion‐conducting mechanism and increases accordingly as water content decreases. As the gel gradually dries into a solid‐like film, the electron‐conducting mechanism facilitated by GNs complements the dominance to ensure high conductivity. Notable, the HAGN hydrogel with 50 mg ml^−1^ GNs (HAGN‐50) demonstrated sustained high conductivity over 72 h, and maintained a conductivity of 2.72 S m^−1^ at 36 h. With water content stabilizing, the conductivity trend leveled off, and the HAGN‐50 retained a high conductivity of 1.84 S m^−1^ even after 72 h, significantly outperforming commercial conductive gels. Besides, we also compared the electrical performance of HAGN‐50 and Greentek in the frequency range of 1–10^5^ Hz within 72 h. The results shown in Figure S5 (Supporting Information) indicate that HAGN‐50 performs a higher sustainable conductivity across the entire testing frequency range. As compared with other research in Table S1 (Supporting Information), HAGN hydrogel, used as a flowable conductive gel in EEG recording, demonstrated sustained high conductivity in both initial wet and dried states after 72 h. Figure 3d and Figure S6 (Supporting Information) illustrate the skin contact impedances of various hydrogels under wet and dry conditions, respectively. The skin contact impedance increases accordingly when the hydrogel is dried, which is due to the poor skin contact caused by water evaporation. Compared with commercial conductive gels, the skin‐contact impedance of HAGN‐50 is significantly lower than that of HAGN‐0 and commercial gels in both wet and dry states at 1 kHz (Figure 3d). The electrical results indicate that the dual‐mode conducting mechanism compensates for the electrical performance after inevitable water loss, hence ensuring long‐term electrical conductivity.

Figure 3e presents the electrochemical impedance spectroscopy (EIS) curves of HAGN hydrogels with different graphite concentrations, the graphite nanoparticle concentrations are 0, 10, 20, 30, 40, 50, 60, and 100 mg ml^−1^ respectively. Considering the potential risk of skin contact,^[^ ^37^, ^47^ ^]^ combined with the EIS results of the HA hydrogels with different ion concentrations (Figure S7, Supporting Information), an ionic hydrogel with a concentration of 6 wt.% NaCl and 6 wt.% KCl (6 wt.% NaCl/KCl) was chosen for subsequent preparation. In contrast to the effect of ionic doping concentration, the graphite doping concentration has a slightly negligible effect on the impedance of the HAGN hydrogels. The cyclic voltammetry (CV) curves of the HAGN hydrogels with different graphite concentrations show that the reduction peak and oxidation peak of the CV curve increase in response to the increased graphite concentrations (Figure 3f), which indicates the enhancement of the electrochemical performance of the HAGN hydrogel. When the graphite concentration reaches 60 mg ml^−1^, the electrochemical performance begins to display a downward trend, which becomes more significant at 100 mg ml^−1^. It is evident that once the doping concentration of graphite reaches a certain threshold, it adversely affects the conductivity of the HAGN hydrogel. Consequently, the ionic concentration and graphite concentration both affect the electrical and electrochemical performance of the HAGN hydrogel. The HAGN hydrogel with a salt ion concentration of 6 wt.% NaCl/KCl and a graphite concentration of 50 mg ml^−1^ was comprehensively selected for further performance characterization and application demonstration. In addition, conductive durability is evaluated by the EIS curve after 1, 10, 100, and 1000 cyclic voltammetry tests, with the results showing stable properties across the entire frequency range of the HAGN hydrogel (Figure 3g).

Furthermore, we investigated the mechanical properties of HAGN hydrogels in both wet and dry states. Flowability ensures conformal adhesion between the conductive gel and the scalp and improves the dynamic adaptability of the gel. Meanwhile, the hydrogel in its dried, solid‐state form requires sufficient tensile strength to maintain stable contact without connection failure or signal deterioration caused by interference factors such as head movement. In a liquid‐like wet state, the HAGN hydrogel acts as an adhesive gel bonding between solid substrates (Figure 3h, left). The maximum adhesive force of the HAGN hydrogel was significantly higher compared to commercial gel (Greentek), demonstrating its considerable adhesiveness (Figure 3i; Figure S8, Supporting Information). The results shown in Figure S8 (Supporting Information) indicate that the intrinsic adhesion properties of HA gels contribute to the considerable adhesiveness of HAGN hydrogel in the wet state, outperforming commercial gels. Besides, comparisons of HAGN with varying GN concentrations show that doping GNs does not significantly affect adhesion in the liquid‐like state. Figure S9 (Supporting Information) presents the contact angle test results of HAGN‐50 and Greentek gel on five different material surfaces. HAGN‐50 consistently exhibits smaller contact angles, indicating superior fluidity and adhesiveness, suggesting that HAGN hydrogel can spread more effectively on substrate surfaces. The HAGN hydrogel also demonstrated considerable flowability and self‐healing properties, as evidenced by the luminosity of the light bulb (Figures S10 and S11, Supporting Information). As shown in Figure S12 (Supporting Information), equal amounts of HAGN hydrogels (HAGN‐50 and HAGN‐0) and commercial gel (Greentek) were applied on a rough sandpaper surface. HAGN‐50 is easier to peel off from the rough surface while maintaining its intact form, indicating higher mechanical strength when dry. Furthermore, the tensile strength of the HAGN film was measured according to ASTM D638 (Figure 3h, right). The results showed that the tensile strength increased accordingly, and the corresponding stretchable range decreased as the graphite concentration increased (Figure 3i; Figure S13, Supporting Information). Consequently, the HAGN hydrogel has excellent fluidity and adhesion in its wet state and exhibits strong tensile strength when dry, comprehensively ensuring a stable and effective connection for long‐term EEG acquisition.

### 2.3 Biocompatibility of the HAGN Hydrogel

**Figure 4:**
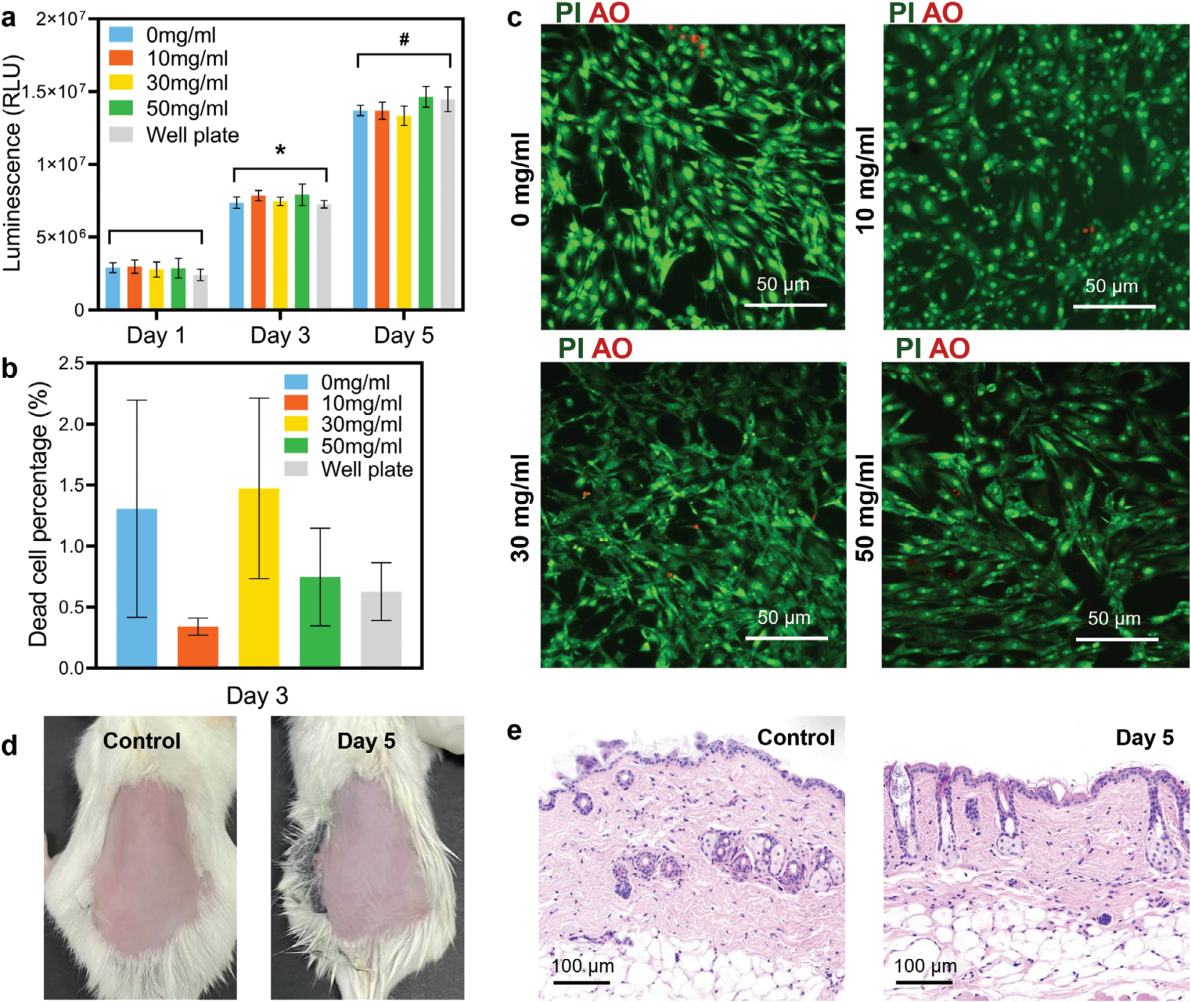
Biocompatibility tests of HAGN hydrogels. a) Cell proliferation results of HAGN hydrogel with different graphite concentrations placed in DMEM complete medium for 5 days. b) Dead cell percentages of HAGN hydrogel groups after 3 days. c) Fluorescence pictures of live/dead cells stained by AO/PI after 5 days. d) Skin irritation test of the skin without hydrogel (left) and after 5 days of HAGN hydrogel (with 50 mg ml^−1^ graphite) application (right). e) Images of hematoxylin and eosin (H&E) stained tissues of the skin without hydrogel (left) and after 5 days of HAGN hydrogel (with 50 mg ml^−1^ graphite) application (right).

Biocompatibility is crucial due to the direct interaction of the HAGN hydrogel with the human epidermis, especially for long‐term EEG recording. To assess biocompatibility, cell viability and skin irritation tests were conducted. Over a 5‐day time frame, L929 cells cultured with the HAGN hydrogel showed significant proliferation in all groups across the time points, with no significant difference among the HAGN hydrogel groups and the control group (**Figure** **4**a). Flow cytometry with propidium iodide (PI) staining revealed that dead cell percentages in all HAGN hydrogel groups were below 3% at day 3, showing no statistically significant deviation from the control group (Figure 4b; Figure S14, Supporting Information). Fluorescence images of acridine orange (AO)/PI‐stained cells on day 5 showed few dead cells (red) among live cells (green) in all HAGN hydrogel groups (Figure 4c), corroborating the low dead cell percentages. These results demonstrate the excellent cytocompatibility of HAGN hydrogels.

Additionally, a skin irritation test was performed on BALB/c mice to further assess the biocompatibility of HAGN hydrogel. Following 5 days of close contact with the HAGN hydrogel, the exposed skin of mice showed no signs of redness, swelling, or exudation (Figure 4d). Detailed examination of the hydrogel‐contact tissue through hematoxylin and eosin (H&E) stained slices revealed no signs of an inflammatory reaction at the application site (Figure 4e; Figure S15, Supporting Information). These findings confirm the highly biocompatible and safe nature of HAGN hydrogel.

### 2.4 Application of the HAGN Hydrogel in In Vivo EEG Monitoring

#### 2.4.1 High Fidelity and Long‐Term EEG Recording

Long‐term, high‐fidelity EEG signal recording is crucial for advancing clinical and research endeavors in non‐invasive neurological applications, including epilepsy studies, sleep monitoring, driving fatigue detection, and human‐computer interaction.^[^ ^48^, ^49^ ^]^ A key factor in maintaining EEG signal fidelity is the impedance between the scalp and electrodes, which directly affects signal amplitude and susceptibility to power line noise.

**Figure 5:**
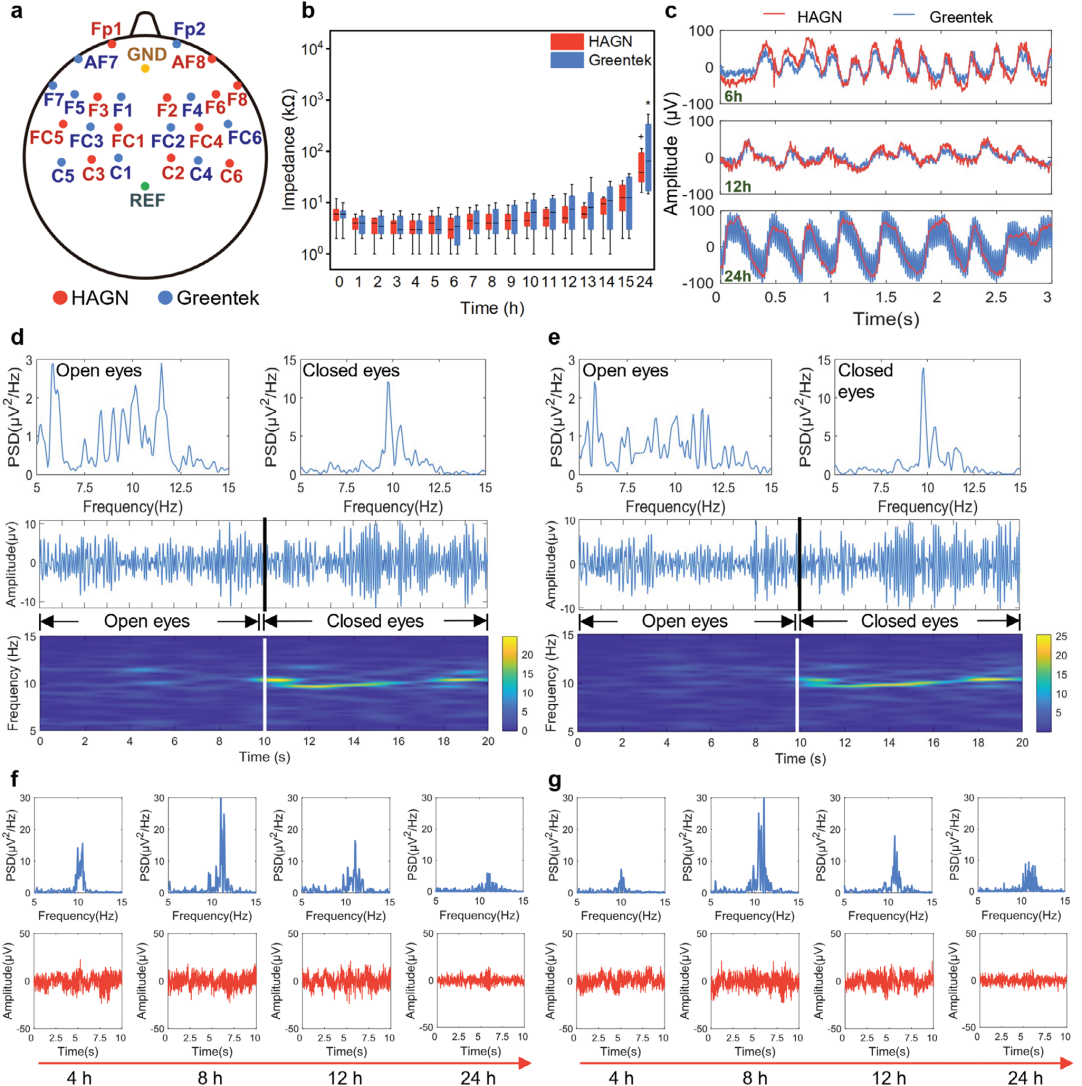
High‐fidelity and long‐term EEG recording on the hairy scalp by HAGN hydrogel and a commercial conductive gel (G5, Greentek). a) Channel locations of 24 working electrodes, a reference electrode, and a ground electrode. b) Impedance variance of the two conductive gels over 24 h of EEG recording. c) EEG signals with eye blinks recorded at channels AF8 (HAGN hydrogel) and AF7 (Greentek gel) at the 6^th^, 12^th^, and 24^th^ hour. d) PSD plots (top), EEG signals (middle), and corresponding frequency spectrogram (bottom) for resting state (open eyes, 0–10 s) and alpha activity (closed eyes, 10–20 s) recorded at channel F2 (HAGN hydrogel) initially. e) PSD plots (top), EEG signals (middle), and corresponding frequency spectrogram (bottom) for resting state (open eyes, 0–10 s) and alpha activity (closed eyes, 10–20 s) recorded at channel F1 (Greentek gel) initially. f) EEG alpha rhythms and their PSD plots recorded by the HAGN hydrogel at channel C3 after 4, 8, 12, and 24 h of continuous wear. g) EEG alpha rhythms and their PSD plots recorded by Greentek gel at channel C4 after 4, 8, 12, and 24 h of continuous wear. Data in b are presented as Mean ± Outlier, with *n* = 12. P values were determined using two‐way ANOVA with Tukey's post‐hoc test. * and + denote significant differences between groups (*p* < 0.05).

To evaluate EEG signal fidelity over time, a healthy subject wore an EEG cap with 24 working electrodes connected to the hairy scalp using either HAGN hydrogel or Greentek gel at symmetrical channels for 12 h (**Figure** **5**a; Figure S16, Supporting Information). The impedance variance across all channels showed no significant difference between the HAGN hydrogel and Greentek gel groups during the initial 15 h. However, at the 24^th^ hour, the HAGN hydrogel group exhibited significantly lower impedance compared to the Greentek gel group (Figure 5b). This trend was also observed in the hairy area (Figure S17a, Supporting Information), but not in the hair‐free area (Figure S17b, Supporting Information), underscoring the superior long‐term conductivity of HAGN hydrogel and its potential for high‐quality EEG recordings.

An initial decrease in impedance during the first 1 to 2 h was likely due to the gradual saturation of the scalp by the gel, enhancing conductivity. Over time, as the conductive gel desiccated, the average impedance increased. By 24 h, both gels showed significantly higher impedance than during the first 15 h. Notably, at 24 h, the HAGN group had more impedance data points below 40 kΩ compared to the Greentek group, indicating the superior sustained conductivity of the HAGN hydrogel (Figure S17, Supporting Information). The threshold of 40 kΩ was chosen based on previous studies demonstrating that high‐fidelity EEG signals are maintained below this level.^[^ ^50^, ^51^ ^]^

EEG waves corresponding to eye blinks were compared between the two gels at the 6^th^, 12^th^, and 24^th^ hours in symmetrical channels (Figure 5c). These recordings revealed the cerebral response to eye blink artifacts, with signal traces showing notable congruence in both waveform and amplitude when impedance was under 40 kΩ. When the impedance of the Greentek gel group exceeded the threshold at the 24^th^ hour, the EEG signals were severely impaired by strong noise interference. Resting state EEG and alpha activity in exemplary recordings from the HAGN hydrogel group (Figure 5d) and the Greentek gel group (Figure 5e) were analyzed, revealing similarities in open and closed eye paradigms. The power spectral density (PSD) plots and spectrogram showed no alpha rhythm during the open eye state and a conspicuous alpha rhythm at 10 Hz during the closed eye state. High‐quality EEG signals over 24 h were evidenced by discernible 10 Hz peaks in the PSD plots (Figure 5f,g; Figures S18–S27, Supporting Information), demonstrating the promising potential of HAGN hydrogel for sustained high‐fidelity EEG recording. Remarkably, at the 24^th^ hour, a decrease in efficacy was observed at one point within the Greentek gel group in the hairy area, whereas no such decline was seen in the HAGN hydrogel group (Figure S27, Supporting Information).

#### 2.4.2 The Signal Acquisition of Steady‐State Visually Evoked Potential (SSVEP) and P300 Tests

**Figure 6:**
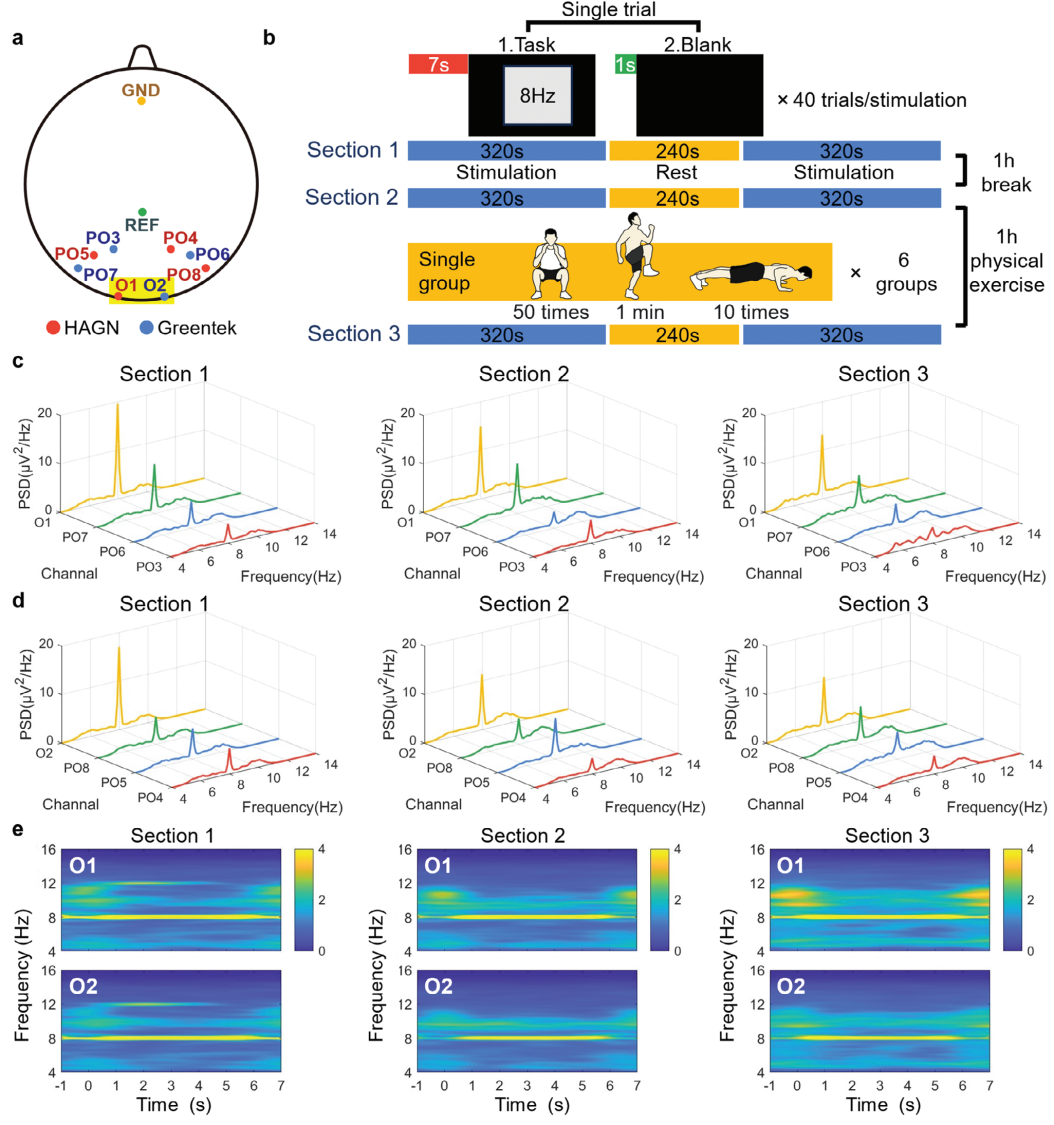
SSVEP protocol and EEG recording on the hairy scalp by HAGN hydrogel and a commercial conductive gel (G5, Greentek). a) Channel locations of 8 working electrodes, a reference electrode, and a ground electrode for SSVEP EEG recording. b) Schematic diagram of the SSVEP protocol and stimulation flow. c) PSD plots for EEG recordings with HAGN hydrogel in 3 sections. d) PSD plots for EEG recordings with Greentek gel group in 3 sections. e) Corresponding frequency spectrograms for EEG recordings with HAGN hydrogel (channel O1) and Greentek gel (channel O2) in 3 sections.

Steady‐state visually evoked potential (SSVEP) and P300 are pivotal in BCI research due to their robustness and reliability, with brain signals exhibiting relatively straightforward patterns compared to other types of brain signals.^[^ ^52^, ^53^, ^54^, ^55^ ^]^ In the SSVEP test, the eight working channels were PO3 to PO8, O1 and O2 (**Figure** **6**a). The SSVEP protocol included three sections to assess signal fidelity after regular activity and vigorous exercise (Figure 6b; Figure S28, Supporting Information). Prominent peaks at the stimulation frequency (8 Hz) were observed in all recorded SSVEPs, evident in the PSD plots, demonstrating the robust SSVEP capture capability of both the HAGN hydrogel (Figure 6c) and the Greentek gel (Figure 6d). The frequency spectrograms for EEG recordings with both gels showed significant signals at the stimulation frequency (Figure 6e; Figure S29, Supporting Information). The signal to noise (SNR) values across the three sections were compared between the HAGN hydrogel and the Greentek gel (Table S2, Supporting Information). No significant differences were observed, indicating comparable performance in EEG signal quality. Additionally, the SNR did not significantly decrease from Sections 1, 2, 3, demonstrating the stability of both gels in signal collection after normal activity or vigorous exercise.

**Figure 7:**
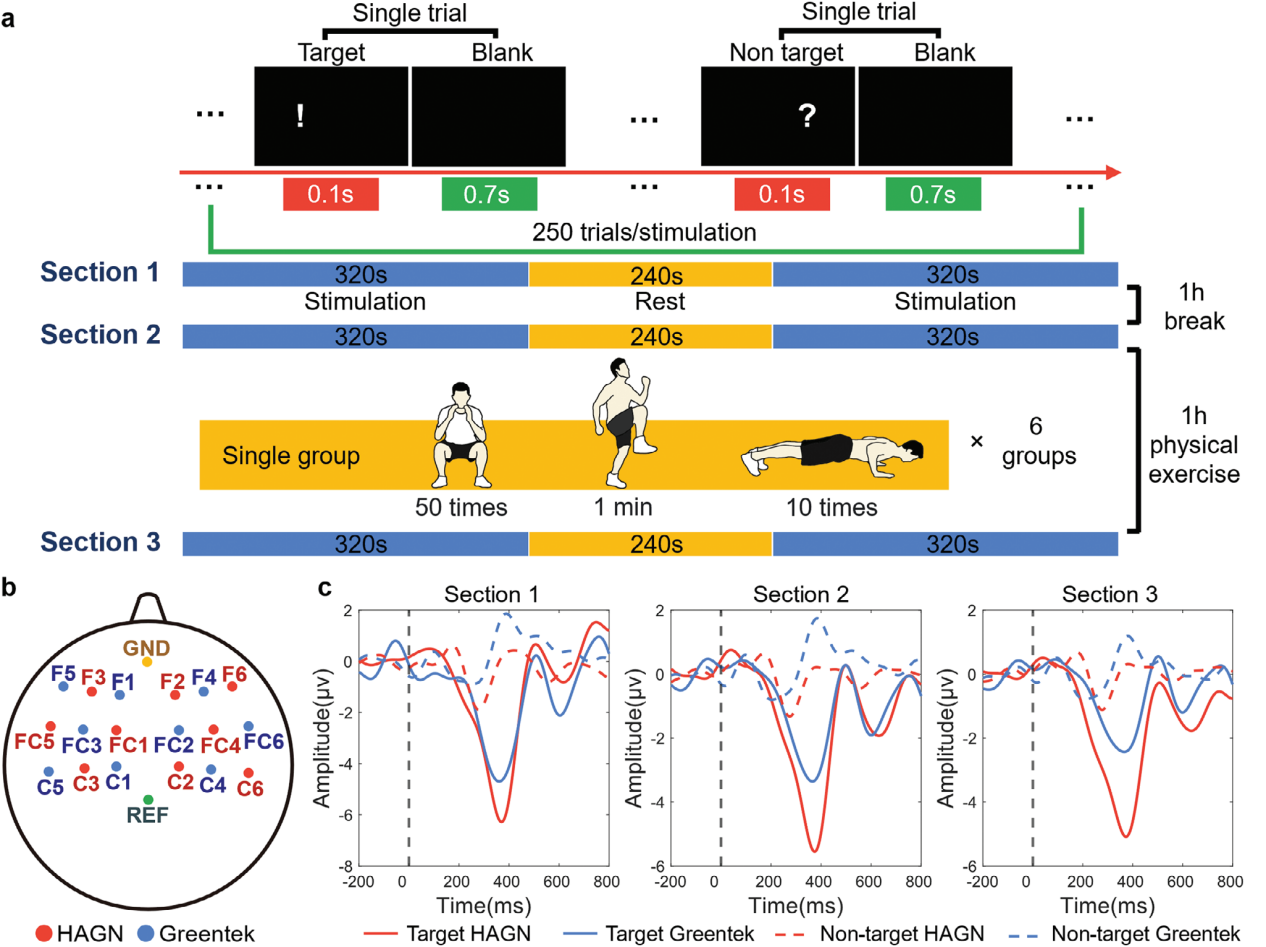
The P300 protocol and EEG recording on the hairy scalp by HAGN hydrogel and a commercial conductive gel (G5, Greentek). a) Schematic diagram of the P300 protocol and stimulation flow. b) Channel locations of 18 working electrodes, a reference electrode, and a ground electrode for SSVEP EEG recording. c) P300 waves at C5 (Greentek gel) and C6 (HAGN hydrogel) channels across 3 sections.

The stimulation flow and visual stimulus images of the P300 test are shown in **Figure** **7**a. Eighteen working electrode positions were selected for analysis (Figure 7b). Upon stimulation, both the HAGN hydrogel and Greentek gel showed a triggered response at ≈300 ms across all three sections (Figure 7c; Figures S30–S32, Supporting Information). The amplitude of the component was initially small, but there was an obvious increase in the amplitude of the peak near 300 ms. Comparing the peak amplitudes ≈300 ms for both gels revealed no significant differences between the gels or among the three stages (Table S3, Supporting Information). This indicates the stability of both gels in capturing P300 signals after regular activity or vigorous exercise. However, due to the superior adhesion and tensile strength of the HAGN hydrogel compared to the Greentek gel, it is plausible that the HAGN hydrogel played a more significant role in electrode stabilization. Further investigations will be conducted to substantiate this hypothesis.

## 3 Discussion

The majority of theoretical and experimental studies on EEG electrode hydrogels have involved sophisticated chemical synthesis. The development of hydrogels for long‐term EEG signal acquisition focuses on conductive stability, mechanical durability, and superior biocompatibility. However, despite the significant research on ionic hydrogels for EEG electrodes, ensuring long‐term conductivity and excellent biocompatibility remains a persistent technical challenge that significantly impacts the quality and practicality of EEG signal acquisition.

In this article, we developed a dual‐mode conductive hydrogel by incorporating electrically active graphite nanoparticles and salt ionic hyaluronic acid to achieve sustained high conductivity. We proposed a straightforward and time‐efficient preparation method with economical natural materials, enabling the possibility of industrial production and biocompatibility. Our work pioneers a breakthrough by breaking the limitation that the inevitable water evaporation has constrained the prolonged conductivity of hydrogel electrodes. This dual‐mode conducting mechanism results from the complementary interaction between ion‐conducting facilitated by salt ions and the electron propulsion, endowing the HAGN hydrogel with prolonged high conductivity under varying moisture conditions. While numerous studies have confirmed the high conductivity of ionic hydrogels, whereas the conductivity is highly dependent on the water content. As water inevitably evaporates over time, it directly affects the conductive instability. Many efforts have been devoted to developing ionic hydrogels that retain water content over extended periods to ensure sustained conductivity, but the practical application suffers complex chemical synthesis and potential toxic hazards. Therefore, introducing an electron‐conducting mechanism offers a practicable solution, complementing the ion‐conducting mechanism to sustain high conductivity properties as the water content decreases. Consequently, we guided the synthesis and preparation of HAGN based on the complementary ion‐electron dual‐mode conducting mechanism to meet the requirements for conductive stability in long‐term EEG signal acquisition.

Furthermore, considering the risks such as skin rashes problems that occur because of prolonged contact of the gel with the scalp during EEG signal acquisition, it is crucial to choose a synthetically safe and non‐toxic gel material with high biocompatibility. Herein, Hyaluronic acid (HA) was employed as the matrix for the conductive ionic hydrogel, which is a naturally occurring linear polysaccharide and widely utilized in creating hydrogel due to its biocompatibility, biodegradability, and ability to retain water.^[^ ^38^ ^]^ Furthermore, the graphite nanoparticles (GN) were selected as conductive additives in the subsequent synthetic preparation due to their excellent electrical performance and biocompatibility.^[^ ^39^, ^56^ ^]^ Based on preliminary experimental results, we optimized the ion concentration ratio in the HAGN hydrogel from the perspective of biocompatibility and mechanical properties, and further optimized the GN doping ratio (Figures S33 and S34, Supporting Information). As a result, the HAGN hydrogels demonstrated excellent biocompatibility, confirmed by cell compatibility studies and skin irritation tests conducted on mice.

Accordingly, the HAGN hydrogel exhibited great potential for long‐term EEG signal acquisition. The HAGN hydrogel was utilized as a conductive medium between the scalp and the EEG cap and performed in vivo EEG test. Comparative analysis with commercial conductive paste showed negligible differences in the long‐term EEG alpha rhythms recorded by HAGN hydrogels, indicating its superior capacity for sustained high‐fidelity EEG recording. Furthermore, representative EEG‐based BCI paradigms, including SSVEP and P300,^[^ ^57^, ^58^, ^59^, ^60^, ^61^ ^]^ demonstrated that the HAGN hydrogels are capable of serving well as a conductive medium in facilitating BCI task execution.

However, further studies are required to further enhance the conductive performance of HAGN hydrogels and validate their potential in practical applications. While the introduction of graphite nanoparticles improves the mechanical properties of HAGN hydrogels, achieving an optimal balance between the effects of doping concentration on adhesion and stretchability remains a challenge. Moreover, the trade‐off between the adhesion and water evaporation requires further investigation. Nevertheless, at the early stages, HAGN hydrogel presents a promising alternative for achieving sustainable high conductivity, mechanical durability, and superior biocompatibility to traditional EEG interface materials.

## 4 Conclusion

In summary, we presented a hyaluronic acid‐graphite nanoparticle ionic gel with stabilized and prolonged high conductivity via a cost‐effective and mass‐producible synthesis method. This complementary ion‐electron dual‐mode conducting mechanism supplements conductivity during desiccation, endowing it with a significant capability for high‐quality EEG signal acquisition. The introduction of graphite nanoparticles enhances conformal contact and provides considerable electrochemical properties. The innovative employment of hyaluronic acid ensures biocompatible application. The HAGN hydrogel demonstrates highly effective and reliable interfaces for the high‐quality capture of long‐term EEG signals, SSVEPs, and P300s, comparable to a commercial gel. The dual‐mode conductive HAGN hydrogel offers a promising engineered‐material strategy for achieving biocompatible, reliable, and stable interfaces in long‐term EEG recording systems and EEG‐based brain‐computer interfaces.

## 5 Experimental Section

### Electrolytic Hydrogel Composition and Preparation

Dispersions of 200‐mesh graphite nanoparticles (Beesley New Materials, Suzhou, China) at specific concentrations were prepared by mixing them with TNWDIS (Chengdu Organic Chemicals, China) and deionized (DI) water. This mixture was stirred at room temperature for 15 min. To further disperse the graphite nanoparticles, a Biosafer 900–92 sonicator (Safer, China) was used at 630 W for 2.5 h in an ice bath. Subsequently, 5% (v/v) glycerin and 2.5% (v/v) 1,2‐propanediol were added to the dispersion while stirring continuously at 500 rpm. NaCl and KCl were then added until fully dissolved. Sifted hyaluronic acid was incorporated into the solution, maintaining the same stirring speed for 2 h. The solute concentrations are detailed in Table S4 (Supporting Information). The samples were sealed and left at room temperature for 24 h to form a semi‐solid hydrogel. The pH of the samples was tested to ensure it was in the neutral range (Table S5, Supporting Information).

### Physical and Chemical Character Investigation

*Scanning Electron Microscope (SEM)*: An SEM (TESCAN MIRA LMS, US) was employed to observe the microstructure of the hydrogels in their dry state. The lyophilized samples were affixed to the sample stage using double‐sided electrical tape, followed by a platinum sputtering process. The morphology of the samples was captured by SEM at magnifications of 100X and 5000X.

*Fourier‐Transform Infrared Spectroscopy (FTIR)*: The chemical structure of the HAGN hydrogel samples was analyzed using FTIR on a Nicolet iS20 FTIR spectrometer (Thermo Fisher Scientific, US). After drying the HAGN hydrogel samples as flat films with a thickness of 0.5 mm, each sample was scanned from 500 to 4000 cm^−1^ for 32 times.

*X‐Ray Diffraction (XRD)*: XRD was employed to assess the crystallinity of the dried HAGN hydrogel samples. After drying the HAGN hydrogel sample (0.1 ml sample^−1^) into flat circular films (diameter = 8 mm), the samples were scanned using an automated multipurpose X‐ray diffractometer (Ultima IV, Rigaku, Japan) with a wavelength of 1.54 Å. Data was collected in the range of 2*θ* = 10° to 80°.

*Raman Spectroscopy*: Raman spectrum was utilized to characterize the chemical component of HAGN hydrogels with/without graphite doping. The HAGN hydrogels without graphite doping and those incorporating graphite at a concentration of 50 mg ml^−1^ were dispensed in 1 ml onto glass slides. Subsequently, the slides were placed on the heating table and subjected to a temperature of 60 °C for 8 h. After the hydrogel samples were dried, a Raman spectrometer (LabRAM HR Evolution, HORIBA scientific, Japan) was used for testing and characterization at a wavelength of 532 nm.

*HAGN Hydrogel Adhesion Strength*: The test of HAGN hydrogel adhesives followed ASTM F2255. It was conducted using an electronic universal testing machine (STS20K, Xiamen Yishite instruments, China), equipped with a 50 N sensor and operated at a test rate of 50 mm min^−1^. The bottom surface of the top mobile platen was covered with the polyimide film. A 1 ml HAGN hydrogel sample was applied to the center of the polyimide plane (diameter = 90 mm), and the adhesive surface had a diameter of 20 mm. After the hydrogel sample contacted the top mobile platen, the top mobile platen was gently moved upward until the bottom polyimide plane reached the specific distance (moving distance = 15 cm). The max separation forces were recorded as the hydrogel adhesion strength.

*Dried HAGN Hydrogel Tensile Strength*: The dried HAGN hydrogel was tested to evaluate the tensile strength. Dried hydrogel samples were shaped as the dog bone piece followed the ASTM D638. The HAGN hydrogel (50 ml) was placed in a 190 mm × 190 mm square dish and dried at room temperature for 72 h to form a dry HAGN film. The film was then cut into test samples measuring 165 mm in length, 19 mm in width, and 5 mm in thickness, with a center bridge length of 50 mm and bridge width of 13 mm. The dog bone pieces were fixed in between two claws of an electronic universal testing machine (STS20K, Xiamen Yishite Instruments, China). The load cell used was 50 N and the extension rate was 50 mm min^−1^. The maximum load (F_max_) at the breaking point was recorded in Newton (N) and then normalized by the cross section area (S_A_) to calculate the tensile strength (R_m_) using the following Equation (1).

(1) $$R_{m}=\frac{F_{max}}{S_{A}}\;$$

*Water Retention Test*: The water retention test assessed the water evaporation ratio of HAGN hydrogels at room temperature. Each 20 ml HAGN gel sample was flattened and placed in a petri dish (diameter = 90 mm), and the initial weight was recorded as “m_0_”. The weights of HAGN gels were then recorded as “m” every hour during the initial 10 h and selected other hours. The final weight of HAGN hydrogels was recorded as “m_c_”. The residual water content at each time point was calculated using the following Equation (2).

(2) $$water\;content\;\left(\%\right)=\;\frac{m-m_{c}}{m_{0}-m_{c}}$$

### Electrical Property Test

*Electrochemical Test in Wet Mode*: Electrochemical impedance spectroscopy (EIS) and cyclic voltammetry (CV) were conducted to assess the performance of functional electrical stimulation and the quality of electrophysiological signal recording of the HAGN hydrogel. An electrochemical workstation (CHI 660D, Chenhua, China) was used to record the EIS and CV graphs. A mold of 9.5 mm × 9.5 mm × 5 mm was filled with the HAGN hydrogel. Two opposite sides inside the mold were affixed with copper foil for attaching measurement electrodes. The frequency range of EIS measurements was set to 10 to 10^5^ Hz, with a voltage of 5 mV. The CV data collection was under the pulse voltage of −0.5 to 0.5 V triangular wave, with a scan rate of 0.01 V s^−1^. The ionic conductivity of hydrogel samples was calculated by the following Equation (3):

(3) $$\sigma \;=\;\frac{L}{R\times S}$$

where L (m) was the distance between two pieces of copper foil, R (Ω) was the intersection of the Nyquist curve at the real part, which was the resistance of the hydrogel at the frequency of 1 kHz, and S (m^2^) was the effective contact area of the hydrogel between two copper foils pieces.

*Skin Contact Impedance*: Skin contact impedance was evaluated using a three‐electrode system with an electrochemical workstation (CHI660D, Chenhua, China). The counter and reference electrodes were commercial electrode pads with attached solid gel (Shenfeng, China). The working electrode was the same electrode pad with HAGN hydrogel or Greentek gel (0.3 ml per sample) after removing the attached solid gel. The reference electrode was placed between the working and counter electrodes when attached to the skin on the inside of the left forearm, ensuring consistent positioning throughout all tests. Impedance was recorded at a voltage of 5 mV across a frequency range of 0.1 – 10 kHz. After drying for 3 days, the dried films of both HAGN and Greentek gels were tested for skin contact impedance under the same voltage and frequency conditions.

### Biocompatible Test

*Cell Viability*: Mouse fibroblast cells (L929) were used to test the cell compatibility of HAGN hydrogels. The salt concentration of HAGN hydrogel samples was diluted 10^3^ times before drying to the solid film. The UV sterilized disc shaped dried HAGN hydrogel films (diameter = 10 mm) were placed in 48 well plates for the evaluation of fibroblasts in 1, 3, and 5 days. After hydrogel films were rinsed in culture media for three times, they were seeded with L929 cells at 10^4^ cells well^−1^. Cells were grown in DMEM media mixed with 10% FBS and 1% penicillin/streptomycin. Cell growth was measured using the Cell Titer Glo luminescent cell viability assay (Promega, US). The assay was based on a luciferin‐luciferase reaction to measure the amount of ATP. Data were reported in relative luminescent units (RLU). Dead cell percentages were calculated by a BD FACSMelody Cell Sorter (BD Biosciences, US). Cell viability and morphology were observed by fluorescent microscopy using acridine orange (AO) and propidium Iodide (PI) stain (Beyotime, China).

*Skin Irritation Test*: To evaluate the biological response of the skin to the HAGN hydrogel, four BALB/c mice (female, 6–8 weeks) were selected for the skin irritation test with one for each time point. The in vivo study protocol was reviewed and approved by the Institute of Radiation Medicine Chinese Academy of Medical Sciences Animal Ethical and Welfare Committee (approval No. IRM‐DWLL‐2023062). After the mice were anesthetized with isoflurane gas, the hair on the back of the mice was shaved by the pet hair clipper (CP‐3380, Codos, China), and the hair removal cream (Veet, Canada) was applied for the further small hair cleaning. Then 0.3 ml HAGN hydrogel of 1 cm^2^ square was applied externally on the bare skin and covered with fixed gauze for 1, 3, and 5 days. After the time points, the gauze was removed, and the exposed skin was carefully observed to check whether it was red, swollen, or seeped. The skin under the HAGN hydrogel was peeled off, fixed with paraformaldehyde, and stained with H&E. Additionally, the unshaved back skin was chosen as a control.

### In Vivo EEG Tests

The EEG signals were recorded using the wireless EEG acquisition system (NeuSen W, neuracle, China) with 60 standard Ag/AgCl electrodes, which were placed on the scalp according to the international 10–20 system. EEG signals were recorded at a sampling rate of 1000 Hz. The EEG study protocol was reviewed and approved by the Tianjin University Office of Research Ethics (protocol No. TJUE‐2021‐019). The HAGN hydrogel was used to provide the conductive pathway between the electrodes of the EEG cap and head skin, as well as secure the electrode position. A commercialized conductive hydrogel (GT5, GREENTEK, China) was used as the control. Both gels were injected into the EEG cap using a syringe to ensure that the contacting impedance between the head skin and electrodes remained below 10 kΩ (Figure S16, Supporting Information).^[^ ^62^ ^]^ The initial threshold of 10 kΩ was selected following the standard of a previous study. After completing the EEG experiments, the gels were removed by washing with water.

*Long‐Term EEG Monitoring*: The long‐term EEG signal monitoring quality was evaluated by a continuous 24 h test. A healthy subject was recruited for this test. The participant wore the EEG cap during the experiment while keeping a normal life except for the data recording time. The impedance between the 24 working electrodes (Figure 5a) and the brain skin was recorded every hour during the initial 15 h and the 24^th^ hour. The waveforms during 1 min of rest with closed eyes, 1 min of rest with open eyes, and 10 s of blinks were collected at the initial 12 h and the 24^th^ hour.

*SSVEP and P300 Protocols*: During the SSVEP and P300 test, the subject sat in front of the stimulation and focused attention on a visual stimulus (Figure S28, Supporting Information). The brainwave signal of the subject was collected through electrodes and amplified by the amplifier, finally transferred to the computer for analyzing the EEG signals. There were 3 sections in each SSVEP or P300 test with 2 stimulation tasks and one rest period after 1 stimulation task. The Section 1 recorded the initial brain signal, while the Section 2 recorded the same brain signal after 1 h of rest of regular normal activity, and the Section 3 recorded the same brain signal after 1 h vigorous physical exercise including 6 groups of 50 times squats, 1 min high‐knees, and 10 times push‐ups.

The SSVEP signal was commonly collected from the occipital lobe area due to its proximity to the visual cortex and free from muscle activity and other sources of electrical noise, further enhancing the quality of SSVEP recordings.^[^ ^63^ ^]^ In the SSVEP test, electrode positions, PO3‐PO8, O1, and O2 were selected for analysis (Figure 6a). The stimulation flow and the visual stimulus picture are shown in Figure 6b. In a single stimulation trial, the strobe lighting lasted for 7 s at a frequency of 8 Hz, followed by 1 s of rest. Each stimulation contained 40 trials.

In the P300 test, electrode positions, F1‐F6, FC1‐FC6, and C1‐C6 were selected for analysis (Figure 7b). The stimulation flow and the visual stimulus picture are shown in Figure 7a. The task design followed the oddball paradigm. Two signals, a target (shape “!”) and a non‐target (shape “?”) randomly appeared in one single trial for 0.1 s, followed by a 0.7 s blank period. Each stimulation contains 250 trials, including 200 non‐target trials and 50 target trials. During the task, the subject was required to count the times of target appearance.

### Statistical Analysis

The characterization of HAGN hydrogels was evaluated with a minimum of three samples per group. Data were presented as mean ± standard deviation (Mean ± SD). Statistical analyses were conducted using SPSS (version 26.0) and GraphPad Prism (version 8.0.0). For experiments involving two variables, a two‐way analysis of variance (ANOVA) followed by Tukey's post‐hoc tests was used. For experiments with a single variable, a one‐way ANOVA followed by Tukey's post‐hoc tests were performed. Differences with p‐values less than 0.05 were considered statistically significant.

### Ethical Approval Statement

Ethical approval for animal surgery in this study was reviewed and approved by the Institute of Radiation Medicine, Chinese Academy of Medical Sciences Animal Ethical and Welfare Committee (approval No. IRM‐DWLL‐2023062). Ethical approval for the electroencephalogram experiment on human participants was reviewed and approved by the Tianjin University Office of Research Ethics (protocol No. TJUE‐2021‐019).

## Conflict of Interest

The authors declare no conflict of interest.

## Author Contributions

H.S. and L.M. contributed equally to this work. H.S. conceived and designed the study, conducted experiments, and contributed to writing and revising the paper. L.M. designed and conducted the electrical and mechanical experiments and contributed to writing and revising the paper. X.C. carried out significant laboratory work. P.L. designed and conducted the EEG experiment, and performed related analyses. J.P. initiated the study. Z.M. conducted the animal study. T.F. provided consultation on the chemical structure and reactions. Y.M. provided consultation on histology analysis. X.M. assisted in the EEG experiment. T.L. supervised the laboratory work and provided funding.

## Supporting information

Supporting Information

## Data Availability

The data that support the findings of this study are available from the corresponding author upon reasonable request.
