# Supplementary material for: A Complementary Dual‐Mode Ion‐Electron Conductive Hydrogel Enables Sustained Conductivity for Prolonged Electroencephalogram Recording
Source: Adv Sci (Weinh). 2024 Aug 8;11(38):2405273. doi: 10.1002/advs.202405273 (PMC11481220; doi:10.1002/advs.202405273)
Supplement: Supplementary file 1 — Supporting Information [file ADVS-11-2405273-s001.pdf]

ADVANCED SCIENCE
Open Access

Supporting Information

for *Adv. Sci.*, DOI 10.1002/advs.202405273

A Complementary Dual-Mode Ion-Electron Conductive Hydrogel Enables Sustained Conductivity for Prolonged Electroencephalogram Recording

*Hengjie Su, Linna Mao, Xiaoqi Chen, Peishuai Liu, Jiangbo Pu, Zhuo Mao, Tomoko Fujiwara, Yue Ma, Xinyang Mao and Ting Li**

Supporting Information

A Complementary Dual-Mode Ion-electron Conductive Hydrogel Enables Sustained Conductivity for Prolonged Electroencephalogram Recording

Hengjie Su[1+], Linna Mao[1+], Xiaoqi Chen[1,2], Peishuai Liu[1], Jiangbo Pu[1], Zhuo Mao[1], Tomoko Fujiwara[3], Yue Ma[1], Xinyang Mao[4], Ting Li[1*]

[1] Chinese Academy of Medical Sciences & Peking Union Medical College, Institute of Biomedical Engineering

[2] Tiangong University, Department of Biomedical Engineering

[3] The University of Memphis, Department of Chemistry

[4] Tianjin Medical University, Department of Biomedical Engineering

[+] They contributed equally in this study

[*] Corresponding author (Email address: liting@bme.cams.cn)

Keywords

conductive hydrogel, electroencephalogram (EEG), dual-mode conducting mechanism, hyaluronic acid, graphite nanoparticles

CONTENT:

Figure

FS1. The fabrication process of HAGN hydrogels
FS2. The XRD patterns of HAGN hydrogels (HAGN-50 and HAGN-0) on a small scale
FS3. SEM images of the HAGN hydrogels with various graphite doping concentrations
FS4. SEM images of the HAGN hydrogels with different salt concentrations
FS5. Waterfall plots of conductivity over time and frequency of HAGN-50 and commercial gel
FS6. Skin contact impedance of HAGN hydrogel and commercial gel
FS7. EIS curves of HA hydrogels with different NaCl/KCl weight ratios
FS8. Maximum adhesive force of the HAGN hydrogels
FS9. Contact angles of HAGN hydrogel (HAGN-50) and commercial gel (Greentek)
FS10. Light bulb illumination with different objects as conductors
FS11. Light bulb illumination before and after cutting and healing of HAGN hydrogels
FS12. Photos of the HAGN hydrogels and commercial gel drop on a rough surface
FS13. The maximum stretching force of HAGN film
FS14. The dead cell percentages of all HAGN groups and the no-gel control group
FS15. H&E-stained tissues of the mouse skin (HAGN-50)
FS16. The appearance of HAGN gel and its application in EEG
FS17. The impedance variance of two conductive gels at the hairy area and the hair-free area
FS18. Power spectral density (PSD) plots of all working channels (0 hour)
FS19. EEG alpha rhythms plots of all working channels (0 hour)
FS20. PSD plots of all working channels (4 hours)
FS21. EEG alpha rhythms plots of all working channels (4 hours)
FS22. PSD plots of all working channels (8 hours)
FS23. EEG alpha rhythms plots of all working channels (8 hours)
FS24. PSD plots of all working channels (12 hours)
FS25. EEG alpha rhythms plots of all working channels (12 hours)
FS26. EEG alpha rhythms plots of all working channels (24 hours)
FS27. EEG alpha rhythms plots of all working channels (24 hours)
FS28. The live images depict the EEG test of the SSVEP and P300 stimulation
FS29. The frequency spectrogram for SSVEP EEG recording
FS30. P300 waves of all working channels in section 1
FS31. P300 waves of all working channels in section 2
FS32. P300 waves of all working channels in section 3
FS33. H&E-stained tissues of the mouse skin (various salt concentrations)
FS34. Mechanical properties of HAGN hydrogels with different salt concentrations

Table

T1. Summary of recent hydrogels for EEG electrodes

T2. The average signal-to-noise ratio (SNR, mean ± std) of SSVEP for the HAGN gel and Greentek gel in sections 1, 2, and 3

T3. The maximum amplitude (μV) of P300 (mean ± std) recorded by the HAGN gel and Greentek gel in sections 1, 2, and 3

T4 Solute concentrations of prepared samples

T5 Representative pH values of prepared samples

Figure

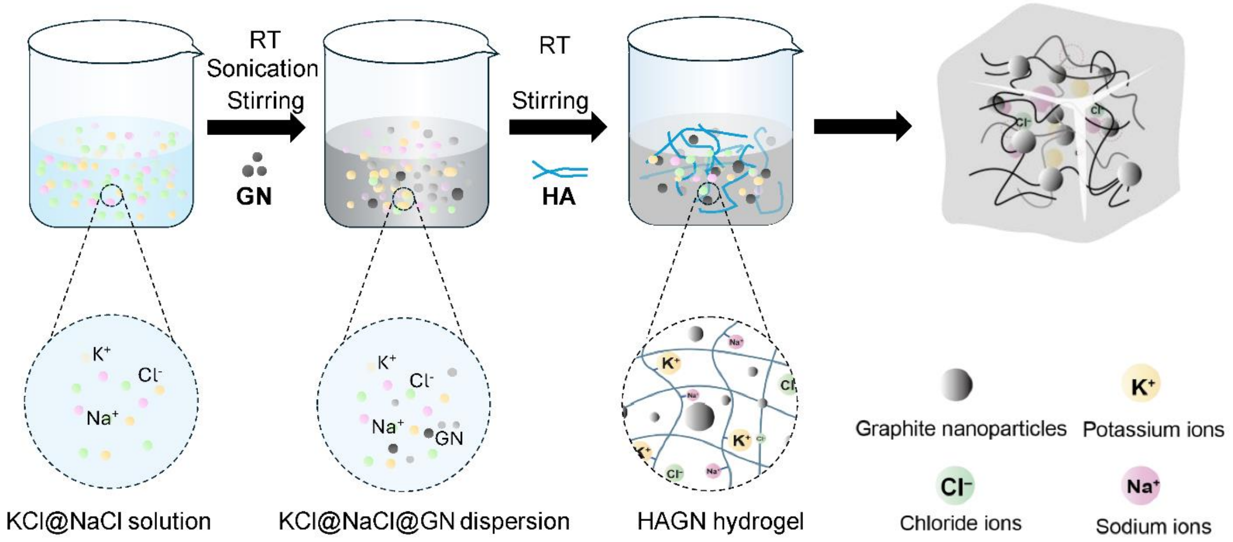

Figure S1. The fabrication process of HAGN hydrogels.

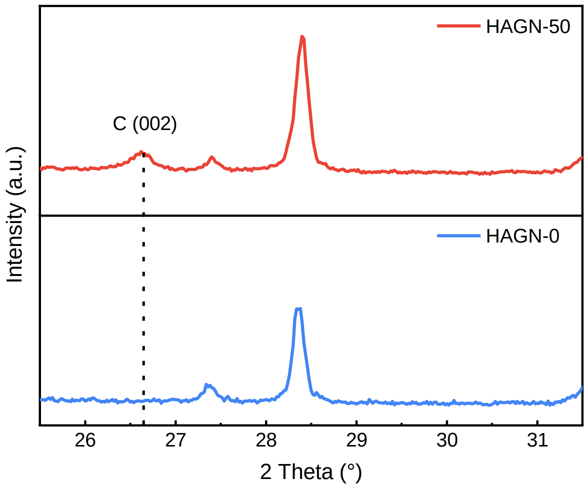

Figure S2. The XRD patterns of HAGN hydrogels with graphite nanoparticles (HAGN-50)/without graphite (HAGN-0) on a small scale.

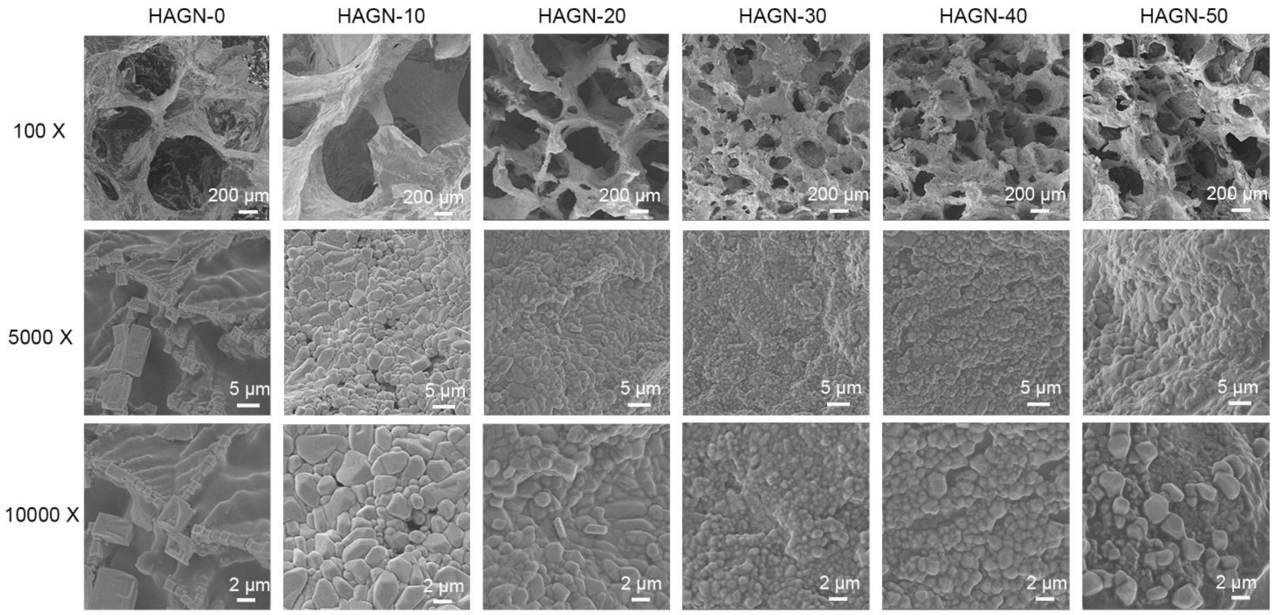

Figure S3. Scanning electron microscopy images of HAGN hydrogels of different graphite concentration and controls at three different magnifications. The salt concentration is 6 wt% NaCl/KCl, the GNP concentrations in HAGN hydrogels are 0, 10 mg/ml, 20 mg/ml, 30 mg/ml, 40 mg/ml, 50 mg/ml, and named HAGN-0, HAGN-10, HAGN-20, HAGN-30, HAGN-40, and HAGN-50, respectively.

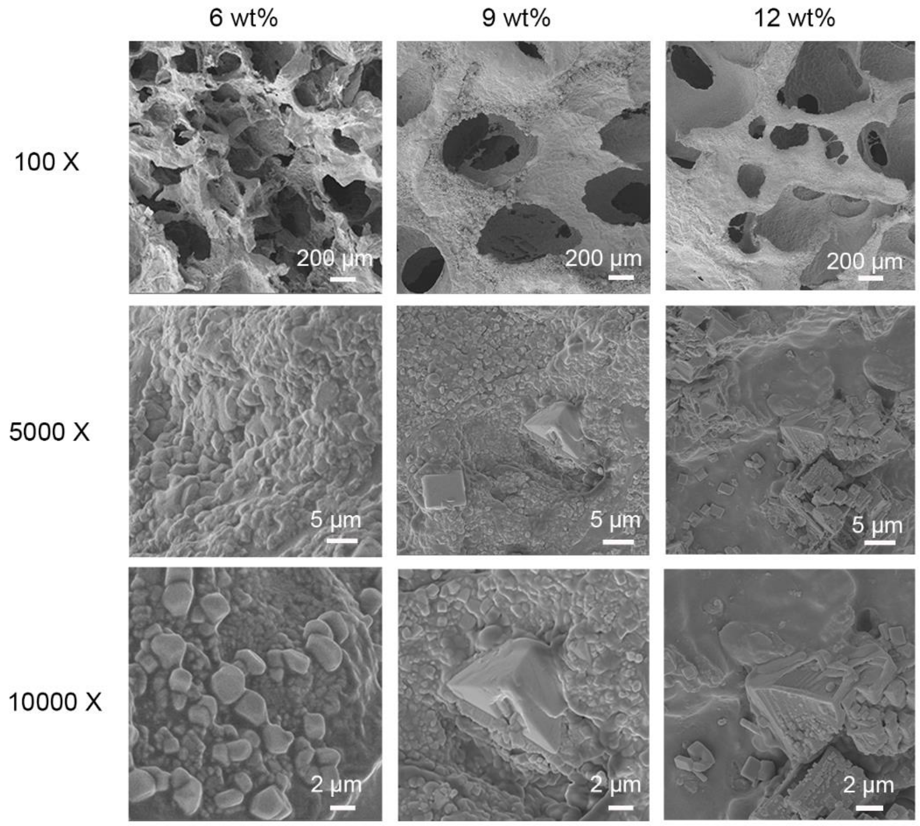

Figure S4. Scanning electron microscopy images of HAGN hydrogels with different salt concentrations and controls at different magnification: The graphite concentration is 50 mg/ml, and the salt concentrations are 6 wt% NaCl/KCl, 9 wt% NaCl/KCl, and 12 wt% NaCl/KCl, respectively.

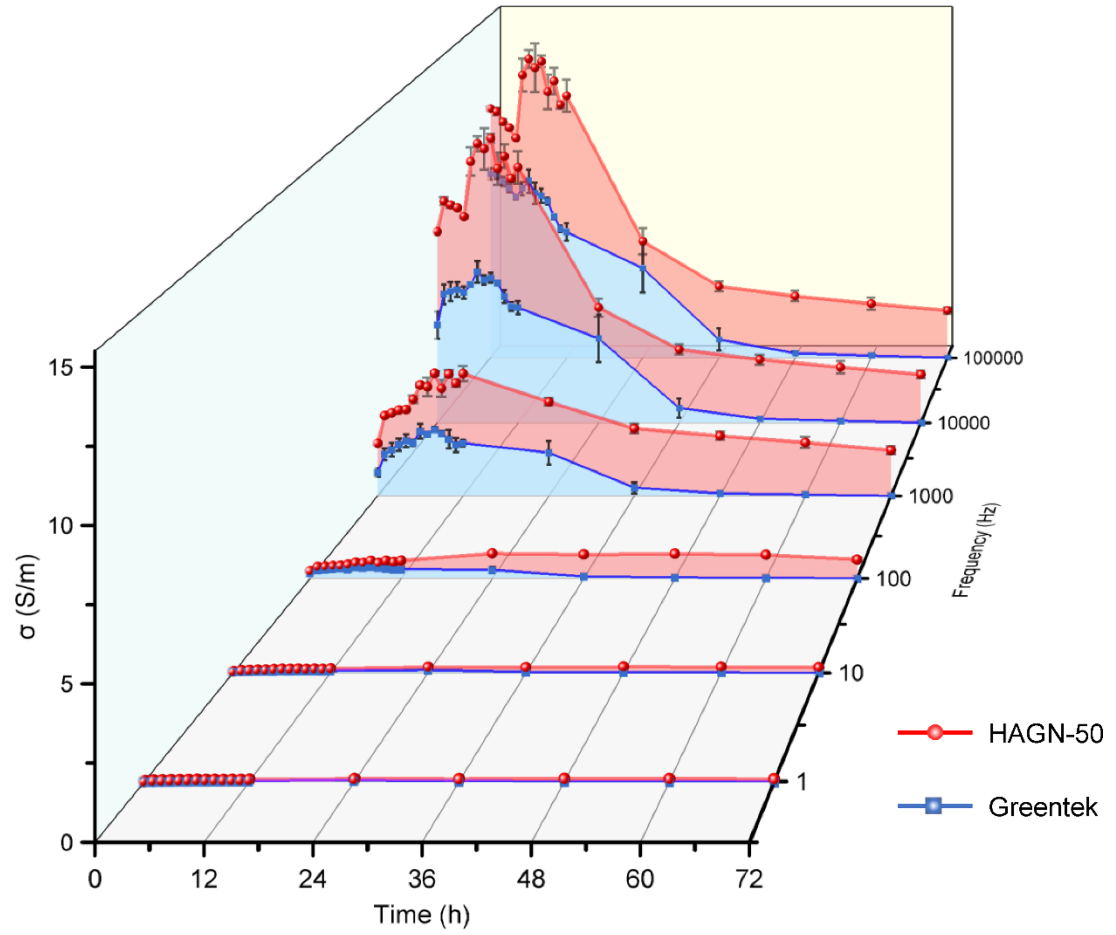

Figure S5. Waterfall plots of conductivity over time and frequency of HAGN hydrogel (HAGN-50) and commercial gels (Greentek). Red: HAGN-50; Blue: Greentek. Data are presented as Mean ± SD, with n = 3.

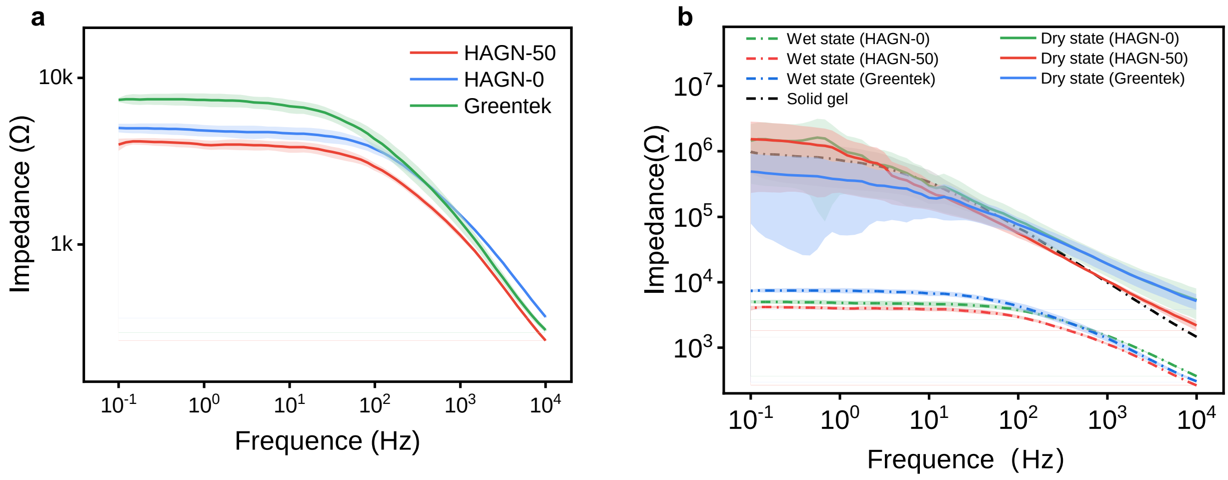

Figure S6. (**a**) Skin contact impedance of the HAGN hydrogels (HAGN-50 and HAGN-10) and commercial gel (Greentek); (**b**) Contact impedance of the HAGN hydrogels (HAGN-50 and HAGN-10) and commercial gels (1. Greentek in wet and dry states, 2. Solid gel of the commercial electrode pad) under wet and dry states respectively. The solid gel is used as a conductive adhesive for commercial ECG patch. HAGN-50 shows lower impedance value than HAGN-10 and commercial gels across the full frequency range in the wet state and lower impedance values in the 10-10k Hz range after natural air drying and curing. The contact impedance performance of the ionic gels (HAGN-50, HAGN-10, and Greentek) in the wet state is better than that of the solid gel. Data are presented as Mean ± SD, with n = 3.

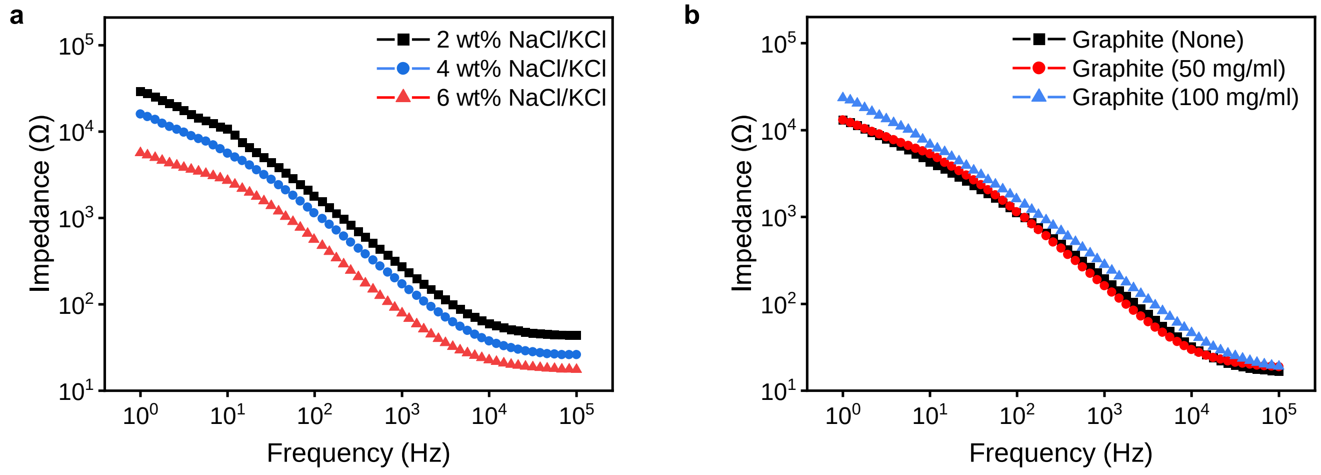

Figure S7. (**a**) EIS curves of HA hydrogels with different NaCl/KCl weight ratios. (**b**) EIS curves of HAGN hydrogels with various contents of graphite with a NaCl/KCl weight ratio of 6 wt%.

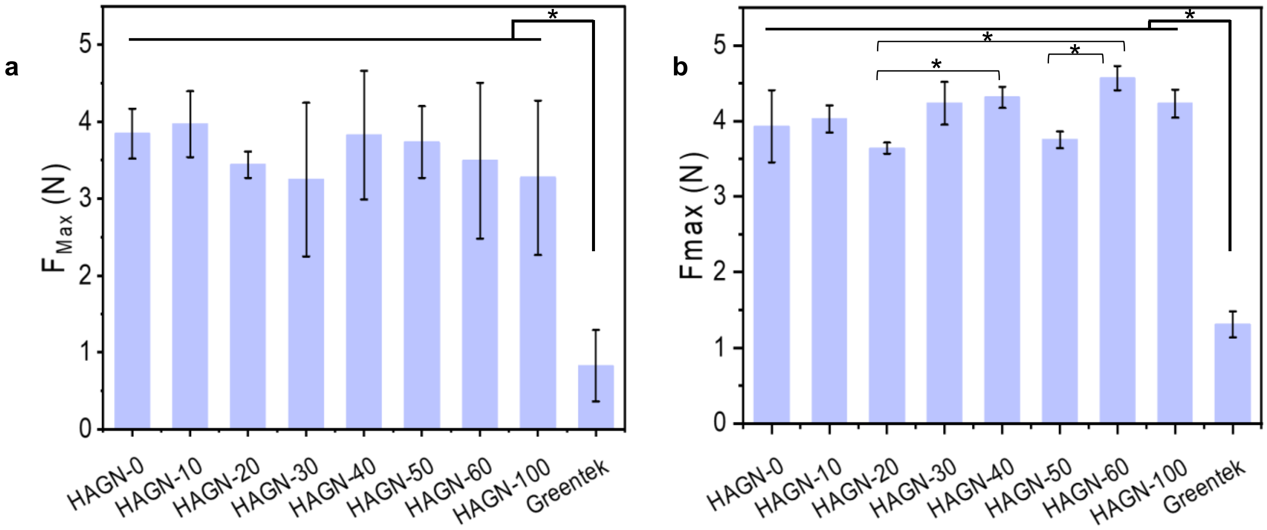

Figure S8. Maximum adhesive force of the HAGN hydrogels with different graphite concentrations in wet condition: (**a**) three samples per concentration were selected for testing; (**b**) each specimen was tested 3 times. Data are presented as Mean ± SD, with n = 3. P values were determined using one-way ANOVA with Tukey's post-hoc test. * denotes significant differences between groups ($p < 0.05$).

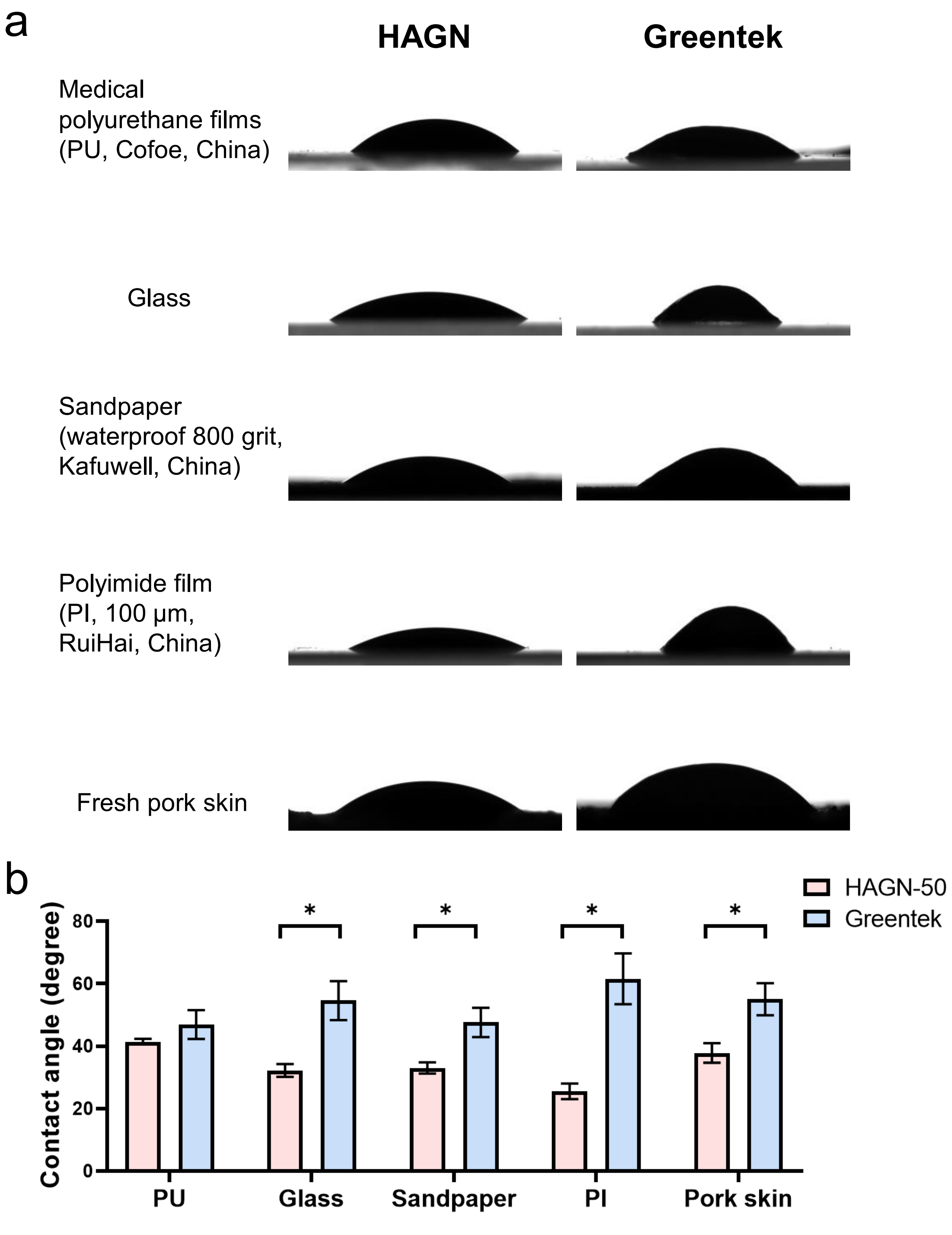

Figure S9. Contact angles of HAGN hydrogel and Greentek gel. (**a)** Images of a single gel drop on various surfaces; (**b)** contact angle measurements. Data in (**b**) are presented as Mean ± SD, with n = 6. P values were determined using two-way ANOVA with Tukey's post-hoc test. * denotes significant differences between groups ($p < 0.05$).

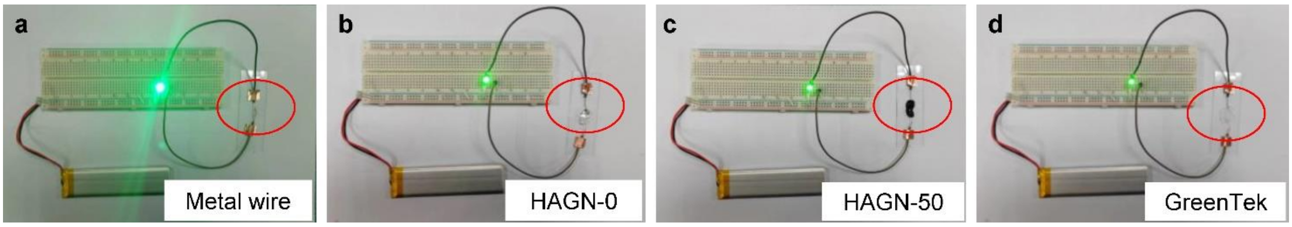

Figure S10. Light bulb illumination with different objects as conductors: (**a**) metal wire; (**b**) HAGN-0; (**c**) HAGN-50; (**d**) Commercial gel (Greentek).

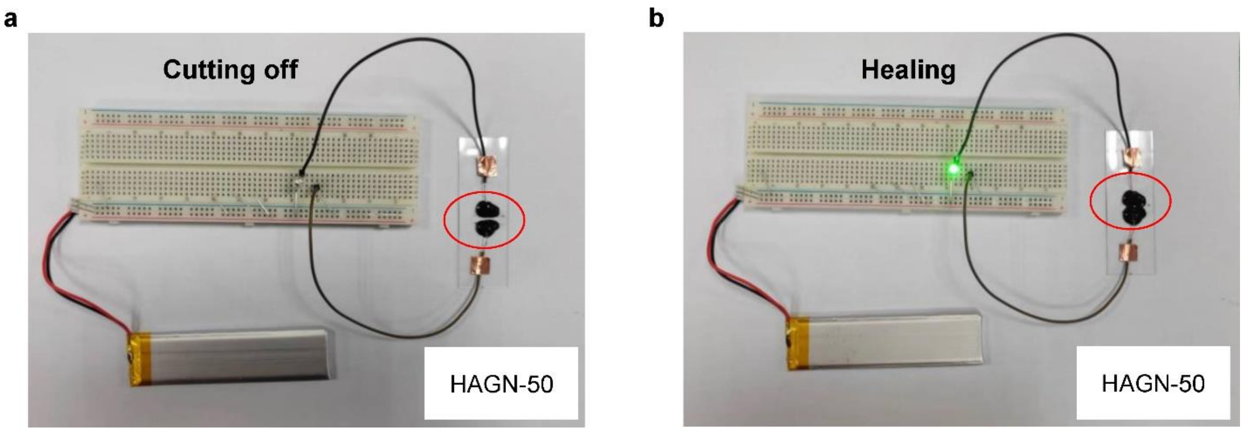

Figure S11. Light bulb illumination:(**a**) when the HAGN hydrogel is cut; (**b**) when the HAGN hydrogel heals after 60 seconds.

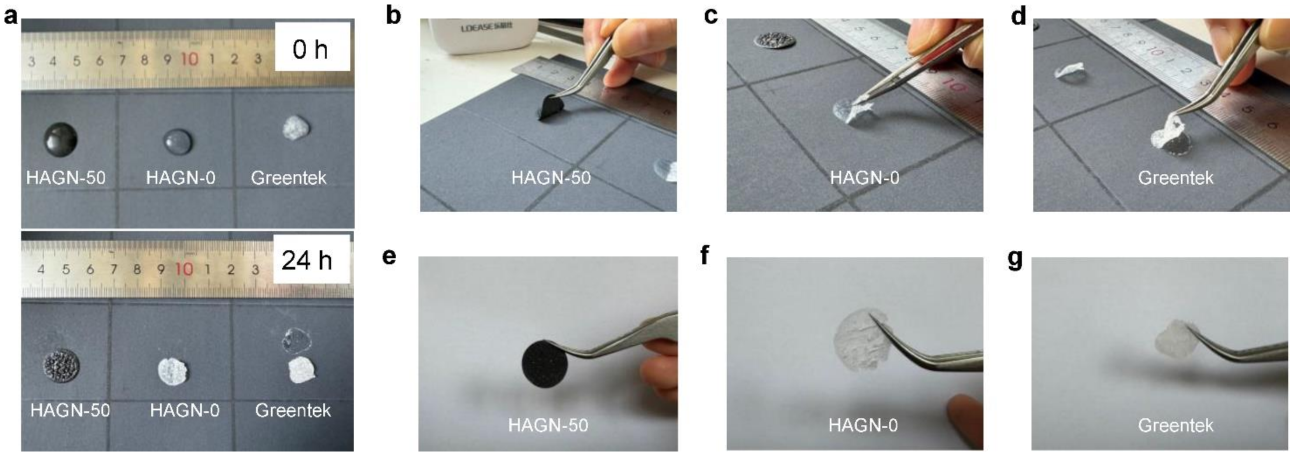

Figure S12. Representative images of HAGN-50 hydrogel, HAGN-0 hydrogel and Greentek gel (0.1 ml each) dried for 24 hours on sandpaper (waterproof 800 grit, Kafuwell, China). (**a**) HAGN hydrogels (HAGN-50 and HAGN-0) and commercial gel (Greentek) dropped on 800 grit sandpaper before and after 24 hours. (**b**)-(**d**) photos of the HAGN films and dried commercial gel under the peeling process; (**e**)-(**g**) photos of the HAGN films and dried commercial gel after being peeled off.

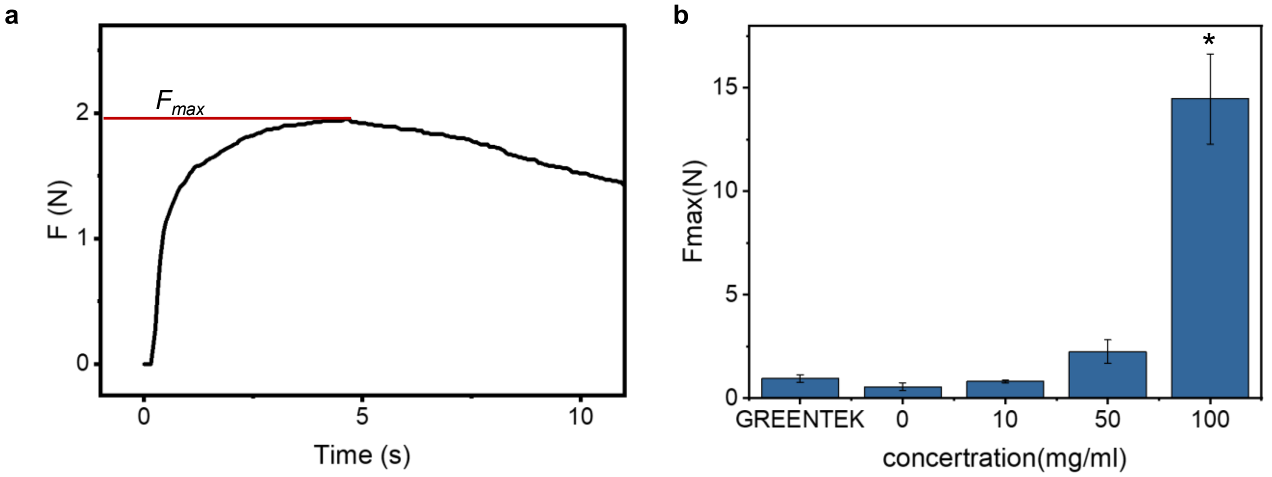

Figure S13. (**a**) The definition of maximum force. (**b**) the maximum force of HAGN film with a water content of 25%, compared with the commercial gel (GREENTEK). Data in (**b**) are presented as Mean ± SD, with n = 3. P values were determined using one-way ANOVA with Tukey's post-hoc test. * denotes significant differences between groups ($p < 0.05$).

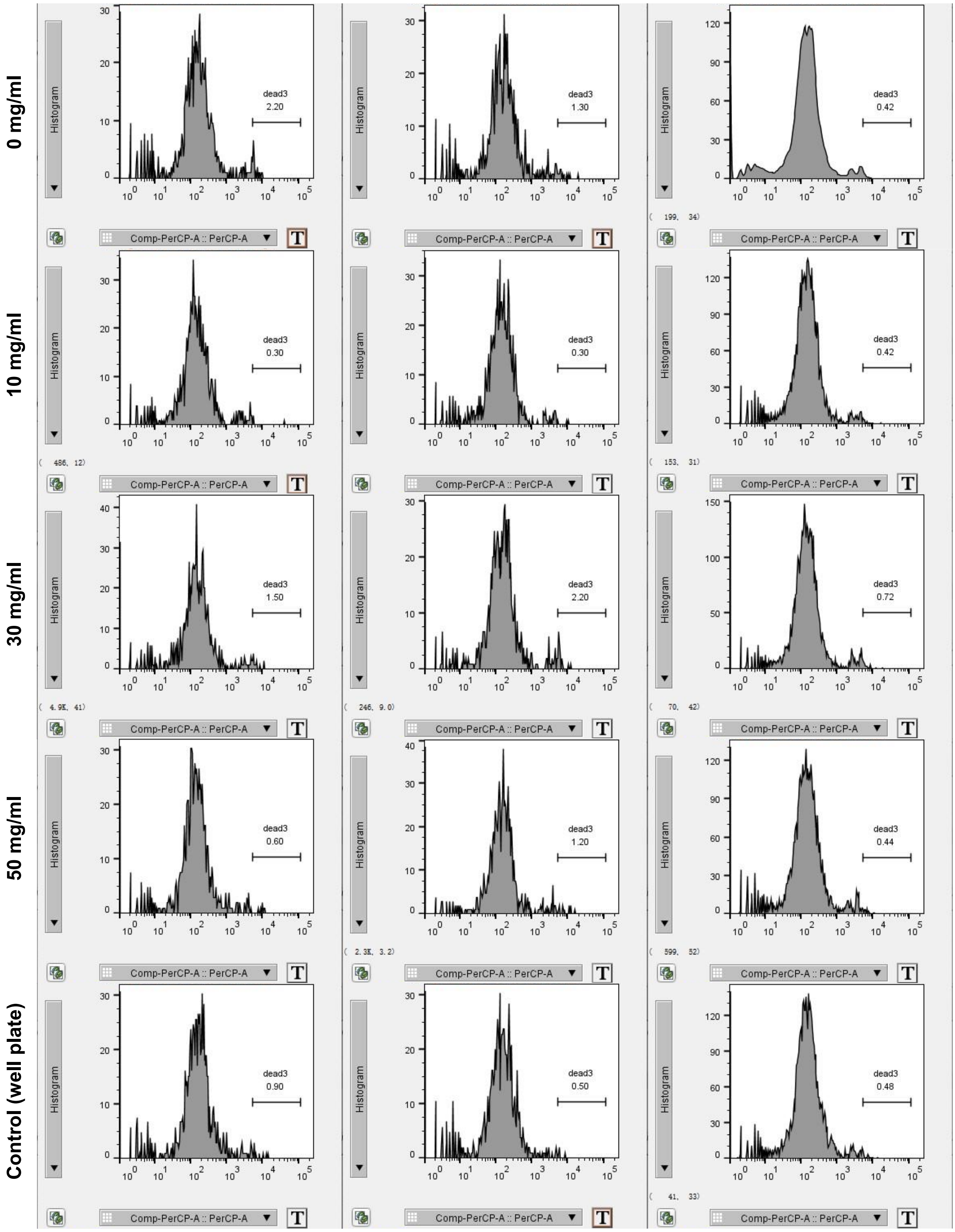

Figure S14. The dead cell percentages of all HAGN groups and the no-gel control, calculated by a cell sorter, were all below 3%.

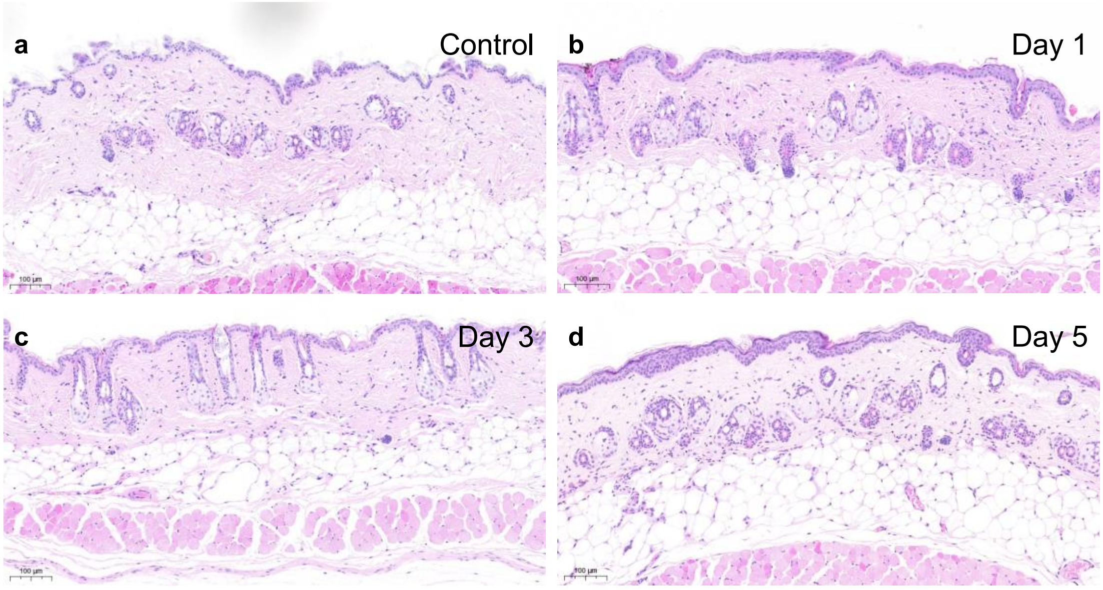

Figure S15. H&E-stained tissues of the mouse skin with (**a**) nothing and with HAGN hydrogel (HAGN-50) at (**b**) day 1, (**c**) day 3 and (**d**) day 5.

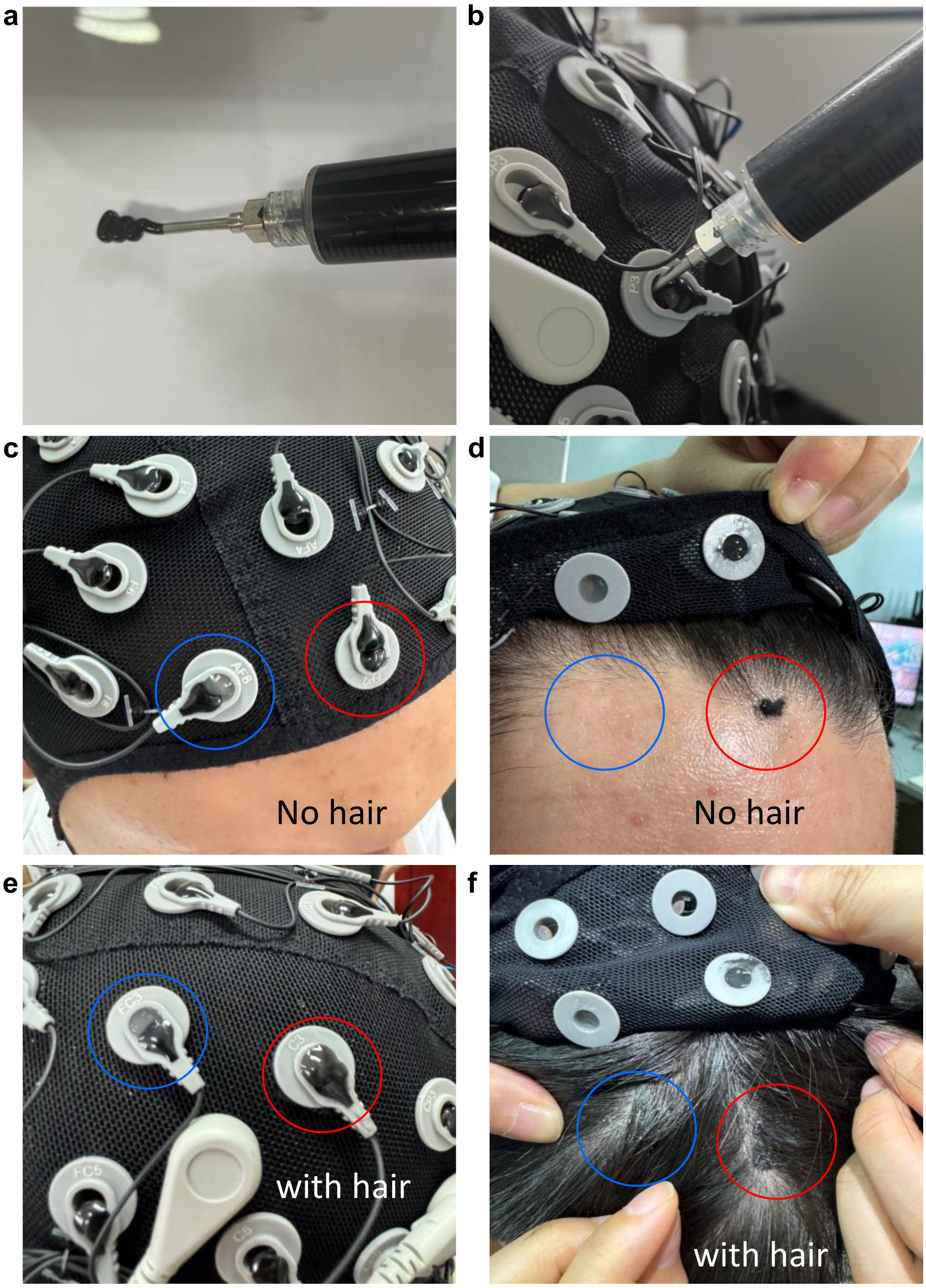

Figure S16. The appearance of HAGN gel and its application in EEG. (**a**) the appearance of HAGN-50 hydrogel; (**b**) the application method of HAGN-50 hydrogel; (**c**) the appearance of applied conductive gels on an EEG cap at hairless positions; (**d**) the appearance of residue gels on hairless skin; **e.** the appearance of applied gels on an EEG cap at hairy positions; (**f**) the appearance of residue gels in hair. In (**c**), (**d**), (**e**), (**f**), the blue circle marks Greentek gel, and the red circle marks HAGN-50 hydrogel.

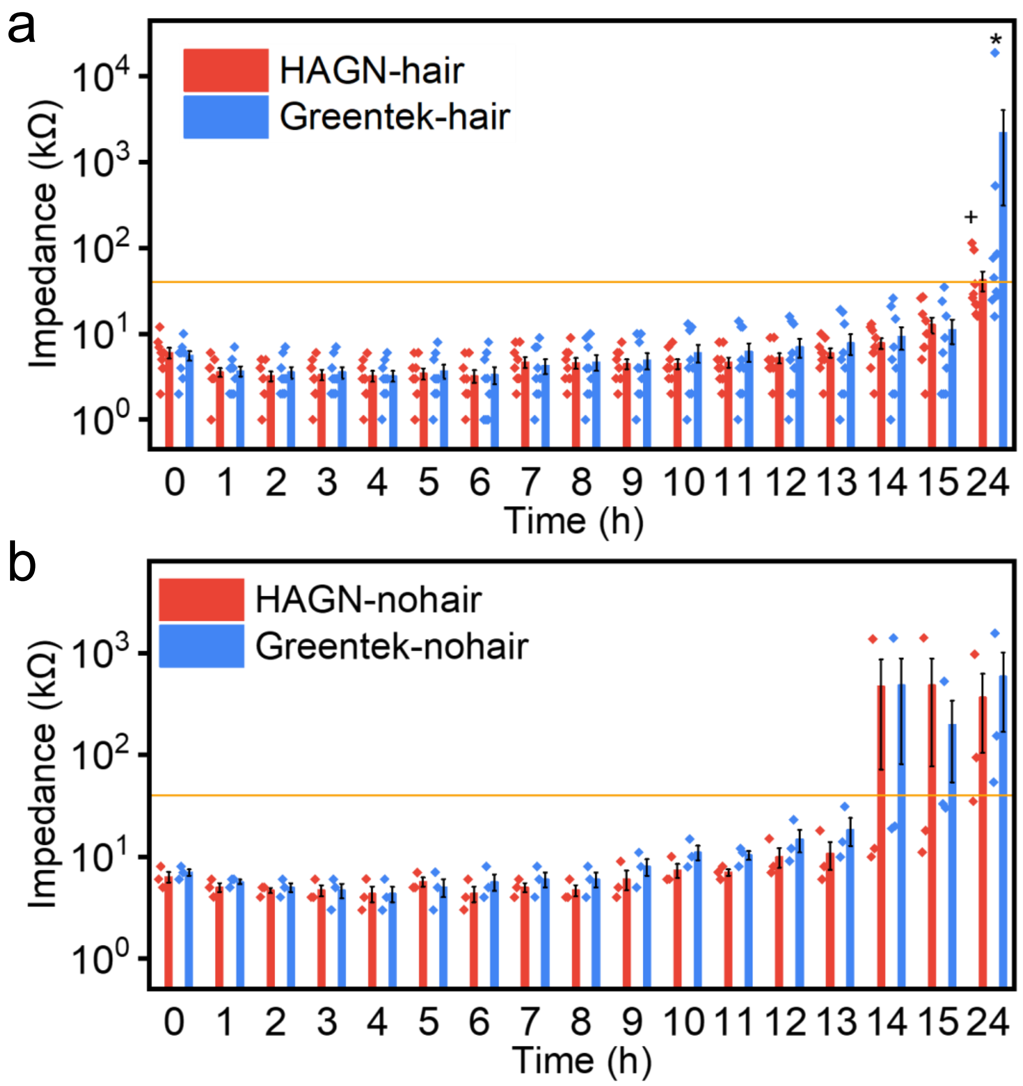

Figure S17. The impedance variance of two conductive gels for 24 hours EEG recording at the (**a**) hairy area and the (**b**) hair-free area. Data are presented as Mean ± 0.5SD, with n = 3. P values were determined using two-way ANOVA with Tukey's post-hoc test. * and + denote significant differences between groups ($p < 0.05$). Yellow lines in the graph indicate the reference line of 40 kΩ.

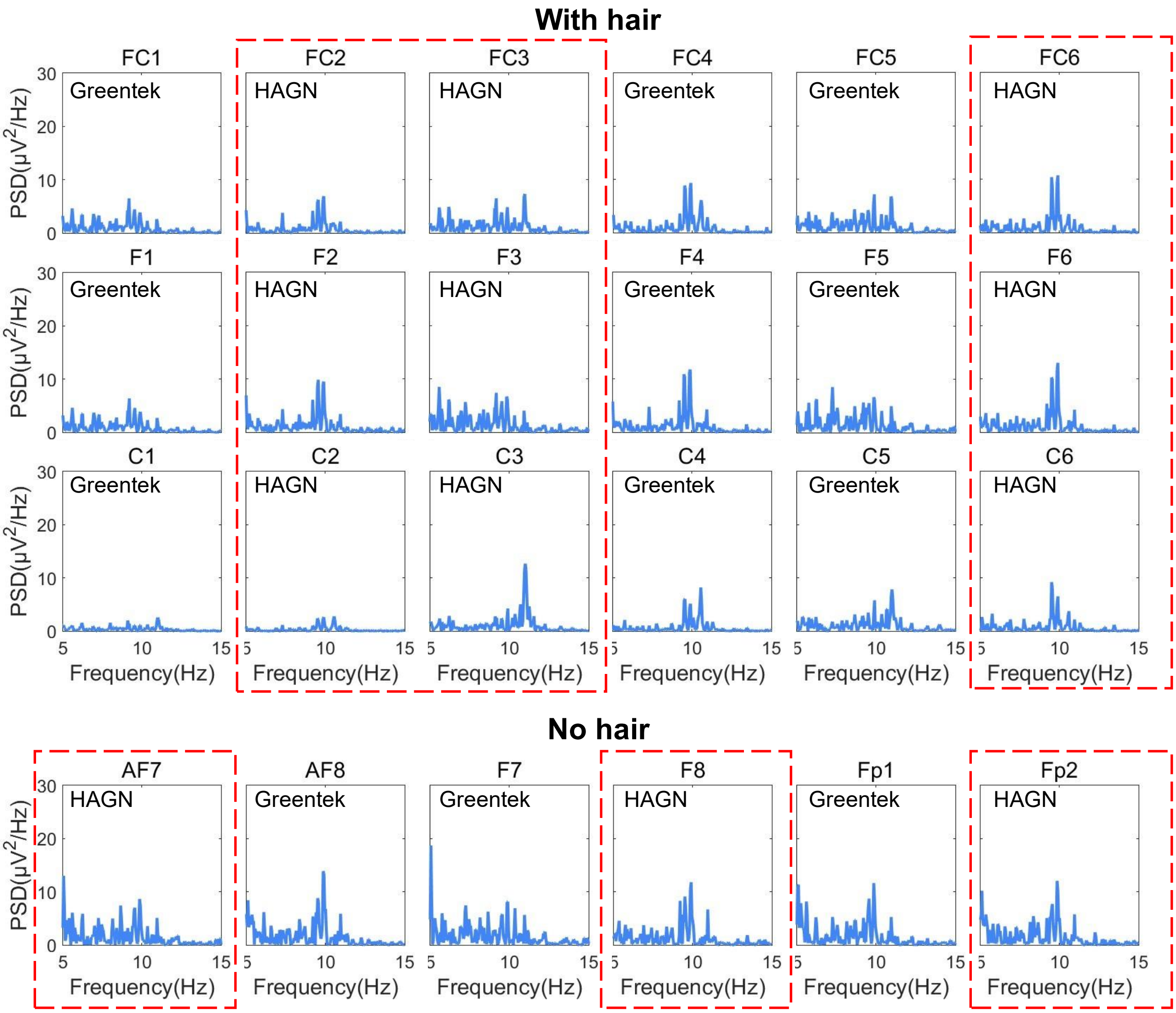

Figure S18. PSD plots of all working channels recorded by the HAGN hydrogel and Greentek gel during closed eyes state at 0 hour. The working channels were segregated into areas with hair and those without.

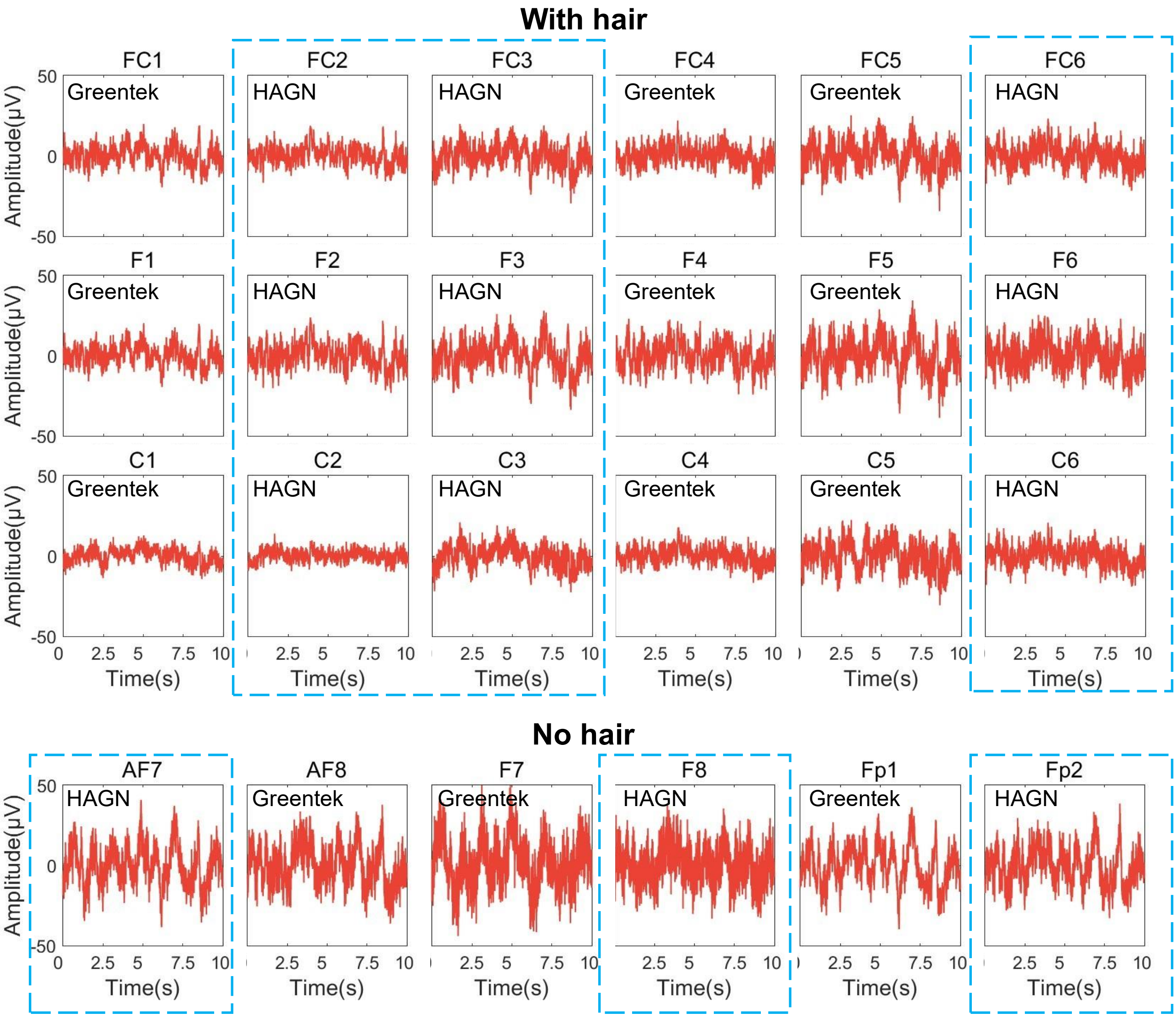

Figure S19. EEG alpha rhythms plots of all working channels recorded by the HAGN hydrogel and Greentek gel at 0 hour. The working channels were segregated into areas with hair and those without.

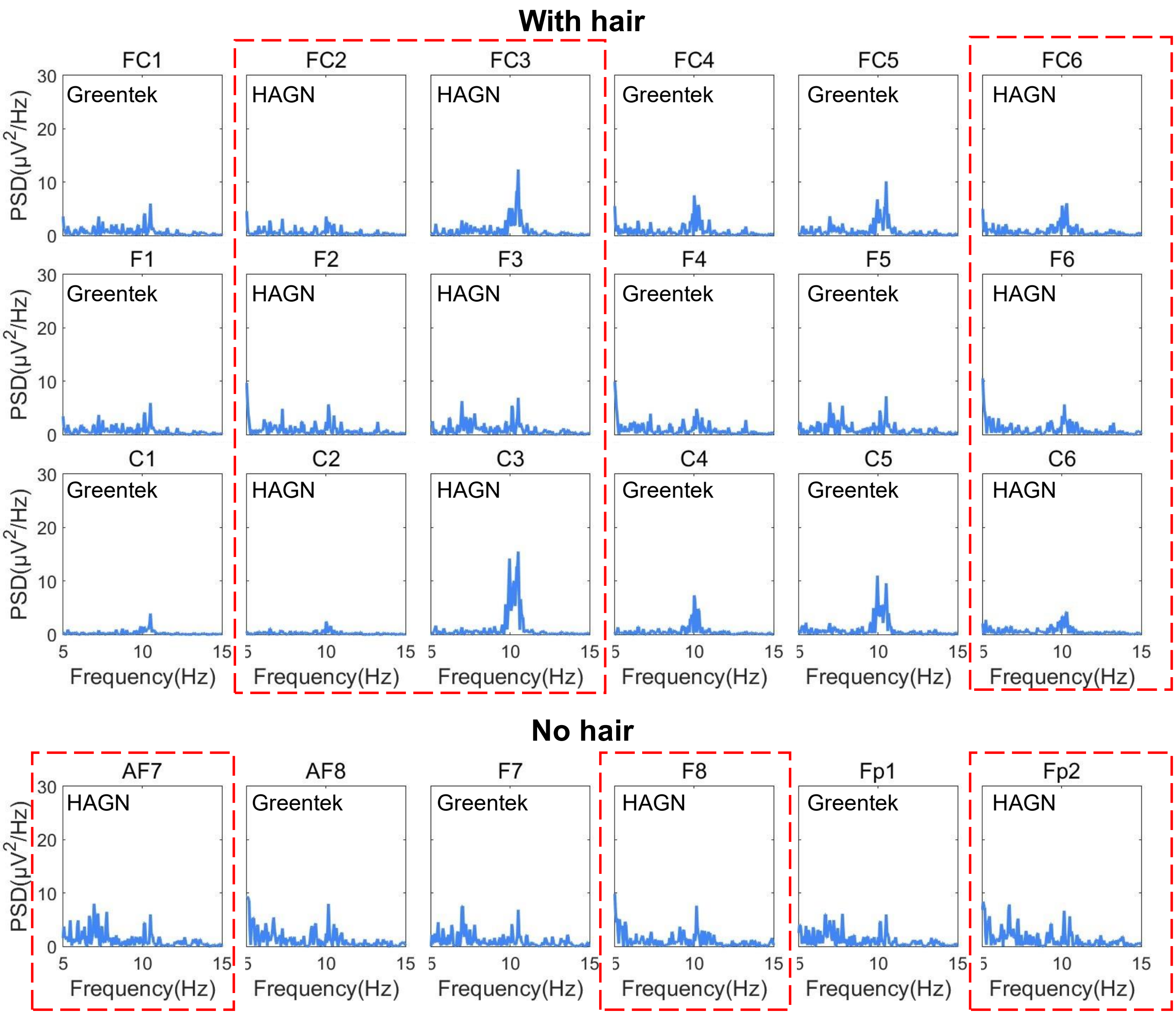

Figure S20. PSD plots of all working channels recorded by the HAGN hydrogel and Greentek gel after continuous wearing for 4 hours during closed eyes state. The working channels were segregated into areas with hair and those without.

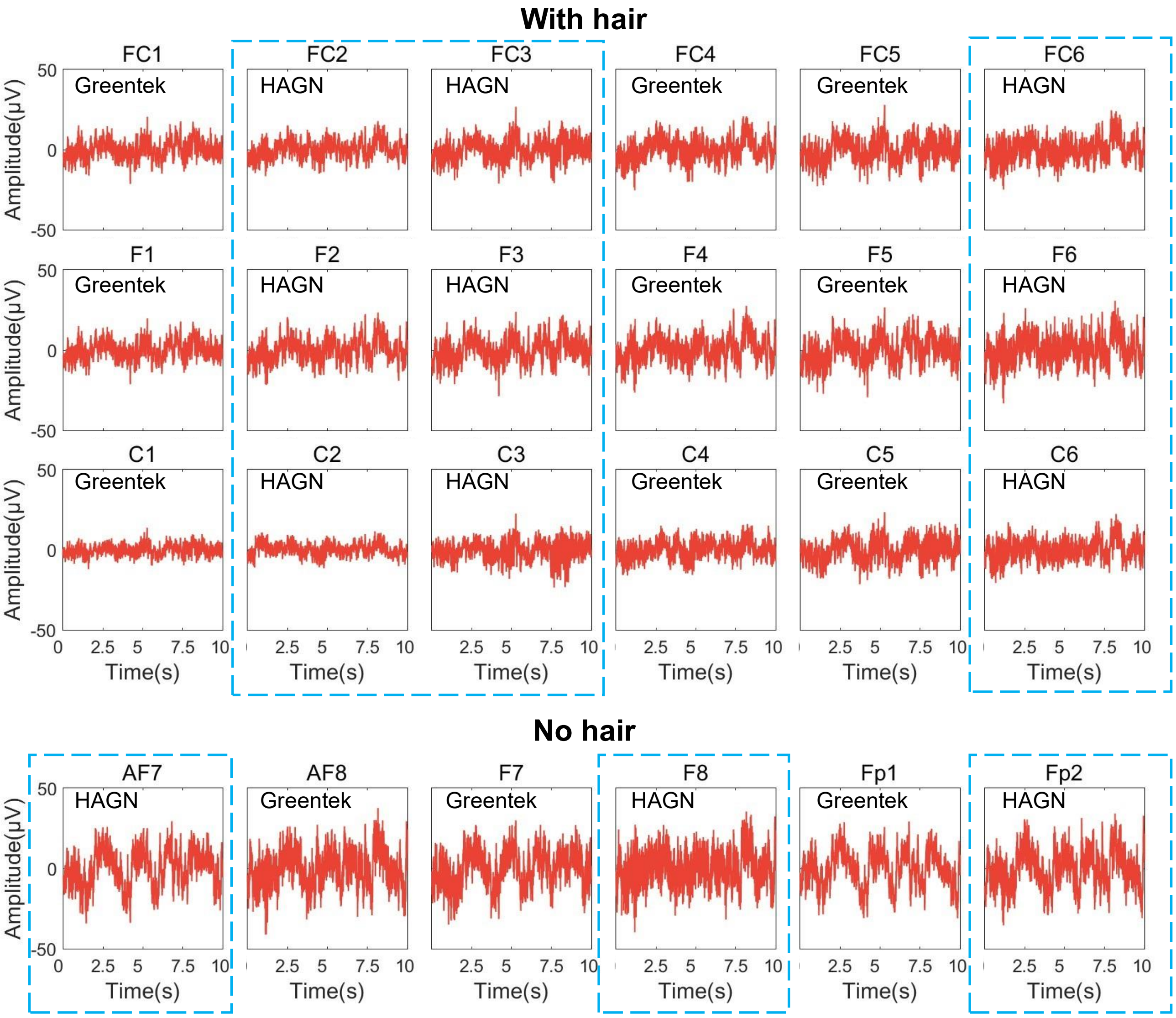

Figure S21. EEG alpha rhythms plots of all working channels recorded by the HAGN hydrogel and Greentek gel after continuous wearing for 4 hours. The working channels were segregated into areas with hair and those without.

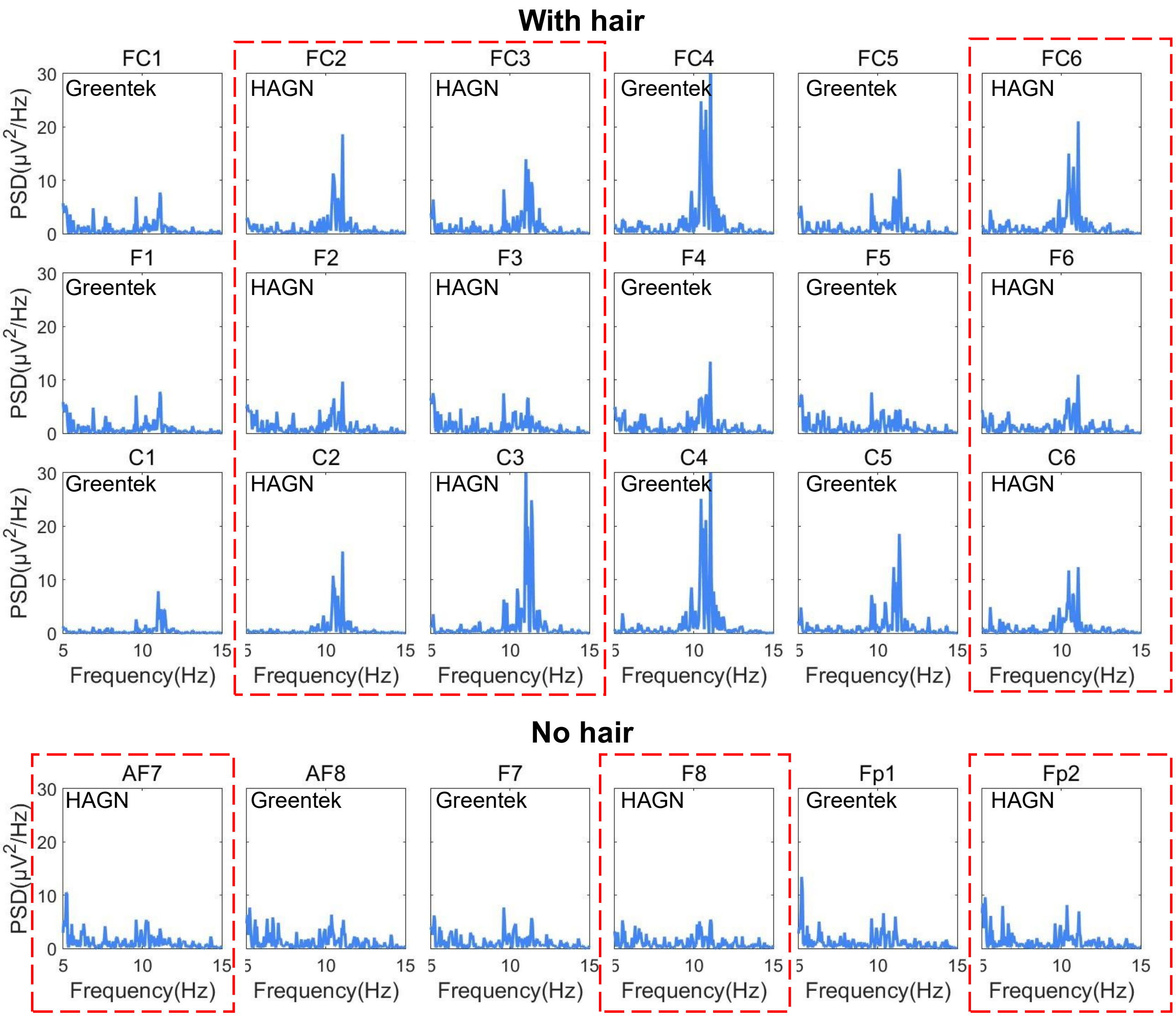

Figure S22. PSD plots of all working channels recorded by the HAGN hydrogel and Greentek gel after continuous wearing for 8 hours during closed eyes state. The working channels were segregated into areas with hair and those without.

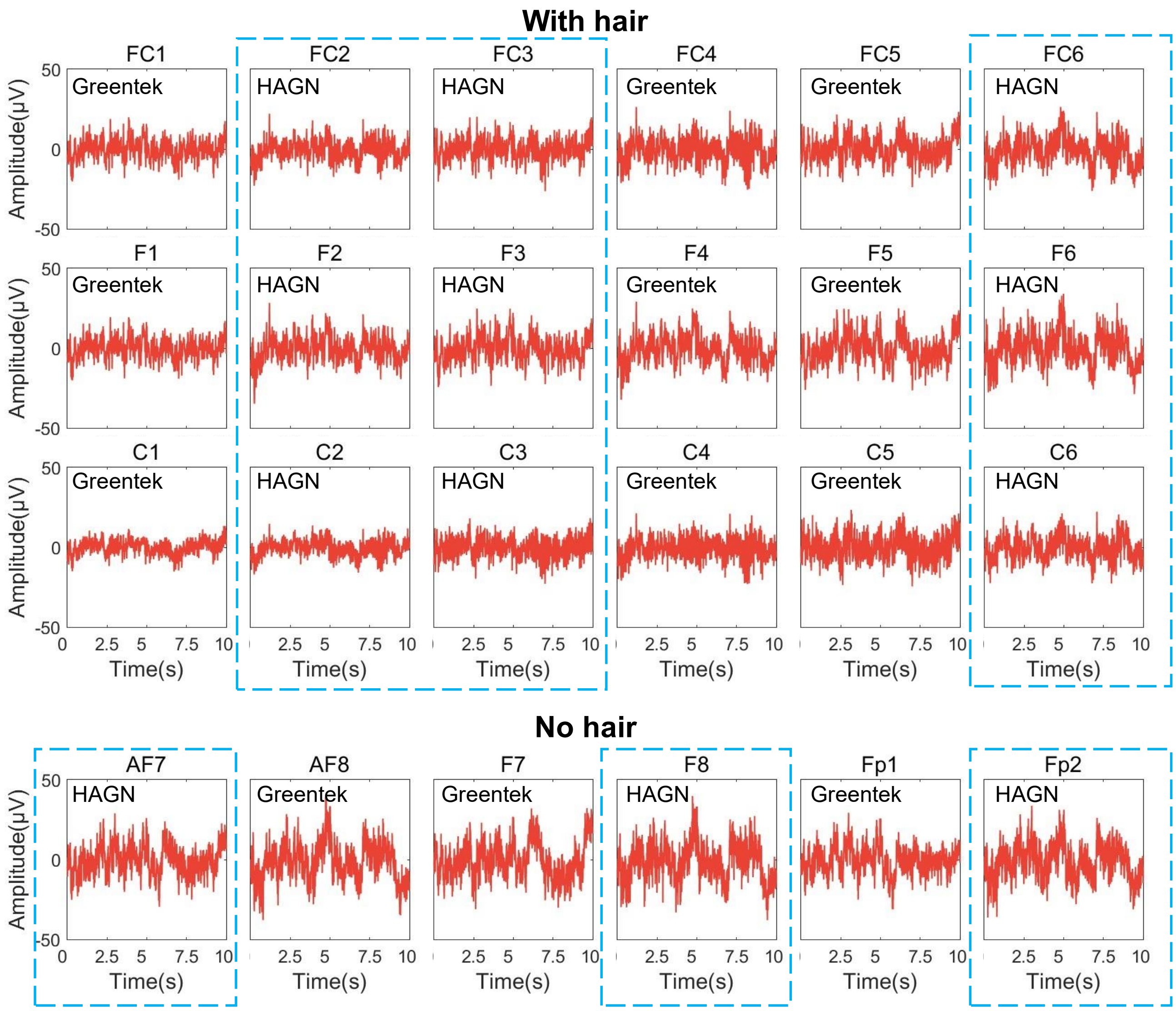

Figure S23. EEG alpha rhythms plots of all working channels recorded by the HAGN hydrogel and Greentek gel after continuous wearing for 8 hours. The working channels were segregated into areas with hair and those without.

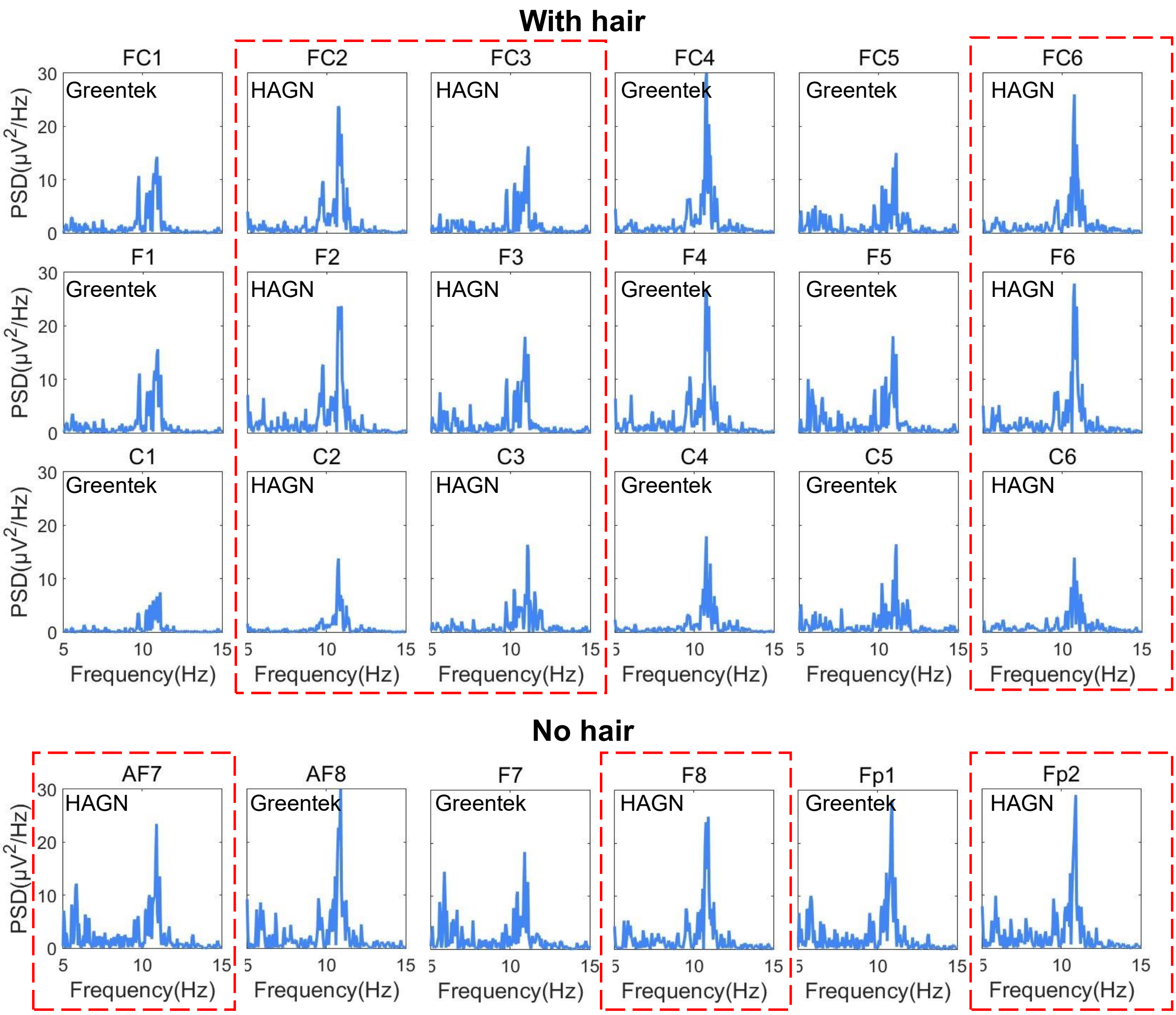

Figure S24. PSD plots of all working channels recorded by the HAGN hydrogel and Greentek gel after continuous wearing for 12 hours during closed eyes state. The working channels were segregated into areas with hair and those without.

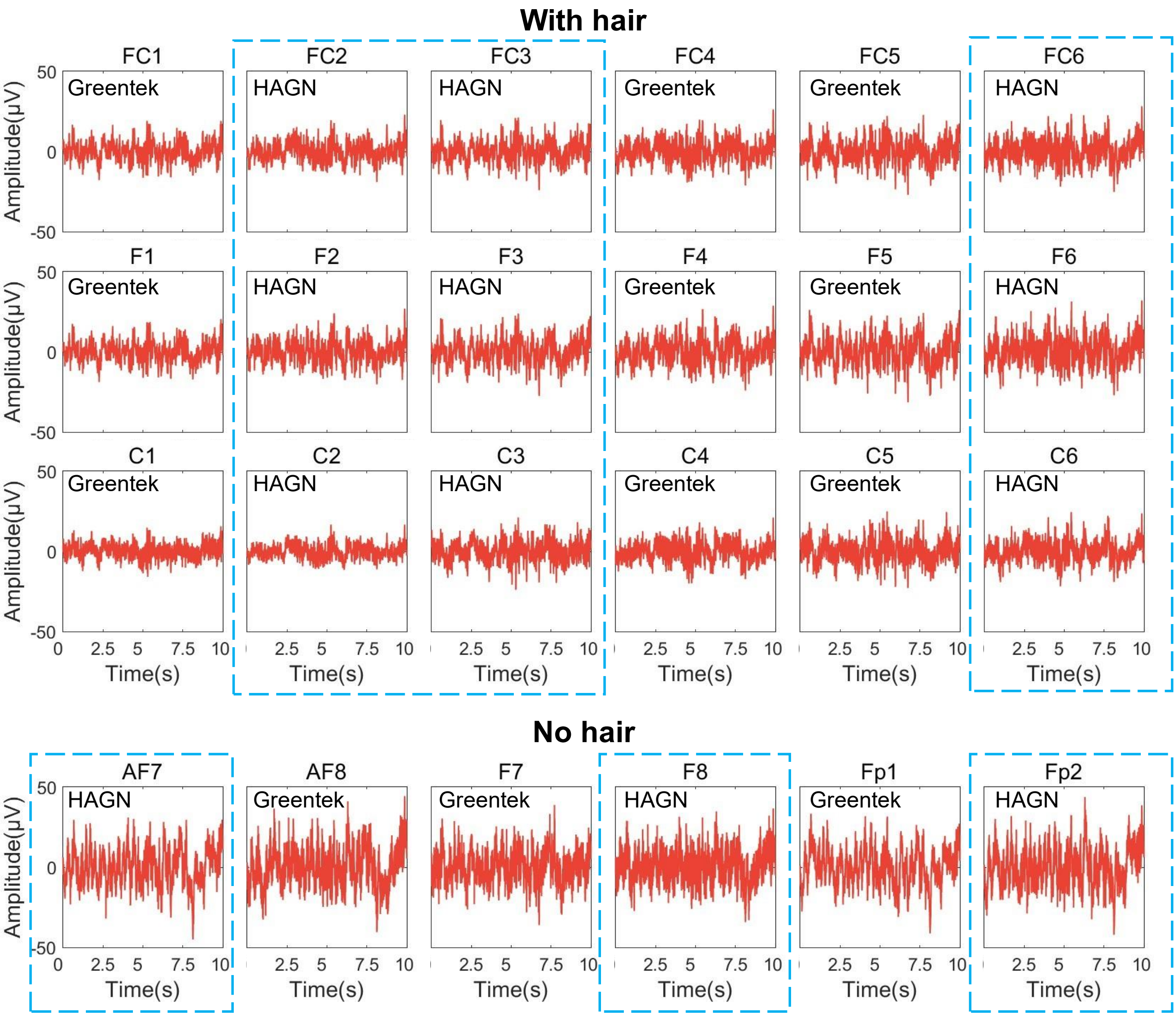

Figure S25. EEG alpha rhythms plots of all working channels recorded by the HAGN hydrogel and Greentek gel after continuous wearing for 12 hours. The working channels were segregated into areas with hair and those without.

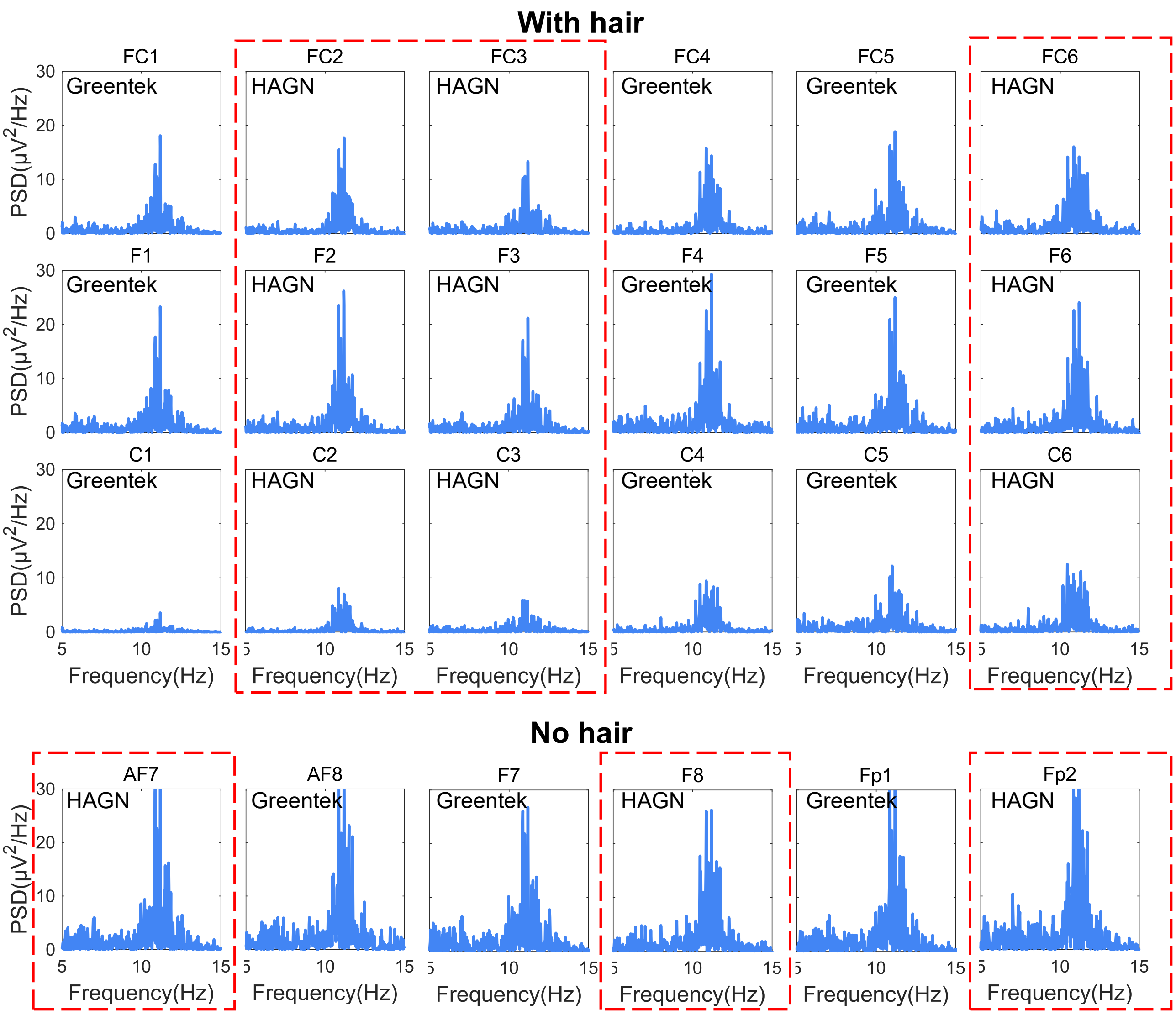

Figure S26. EEG alpha rhythms plots of all working channels recorded by the HAGN hydrogel and Greentek gel after continuous wearing for 24 hours. The working channels were segregated into areas with hair and those without.

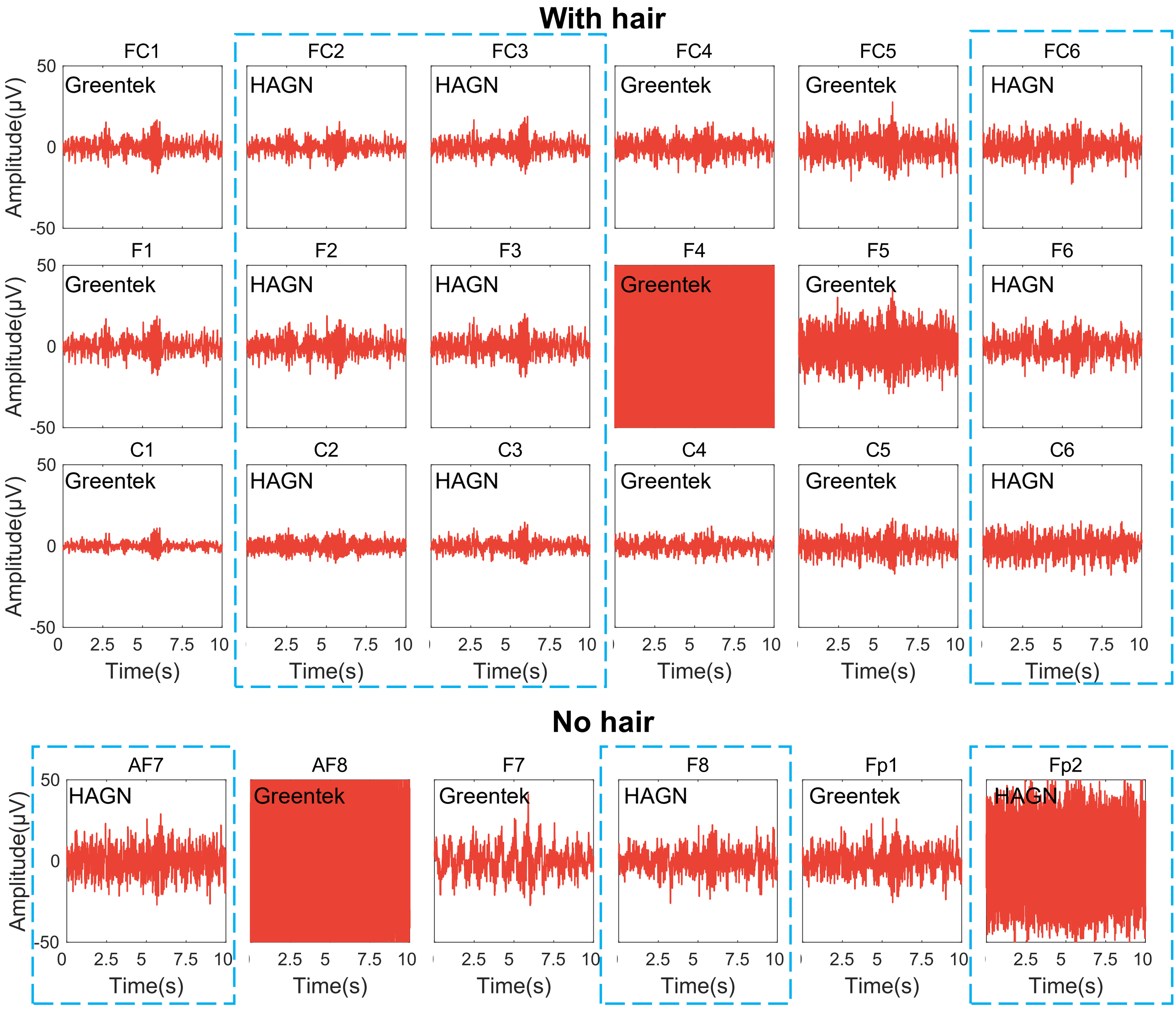

Figure S27. EEG alpha rhythms plots of all working channels recorded by the HAGN hydrogel and Greentek gel after continuous wearing for 24 hours. The working channels were segregated into areas with hair and those without.

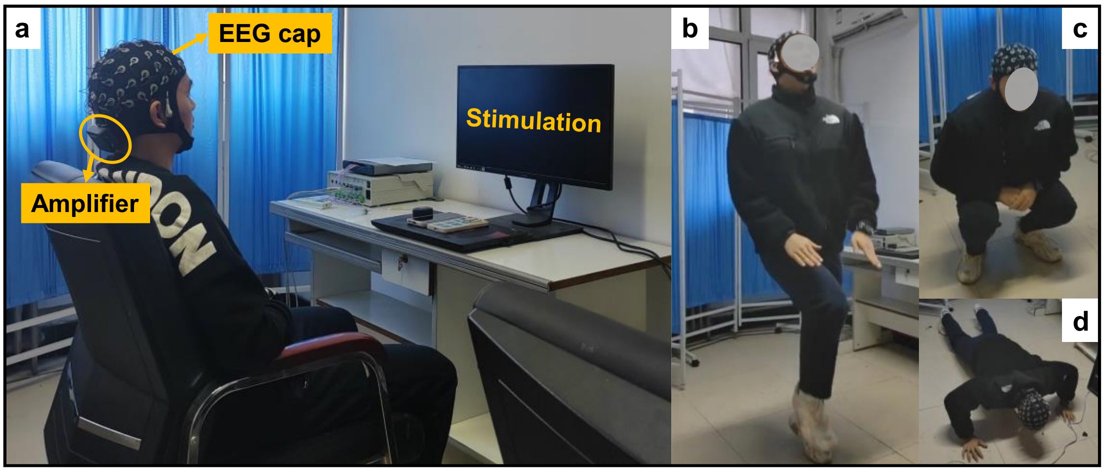

Figure S28. The live images depict (**a**) the EEG test of the SSVEP and P300 stimulation, and physical exercises conducted between section 2 and section 3, including (**c**) squats, (**b**) high knees, and (**d**) push-ups.

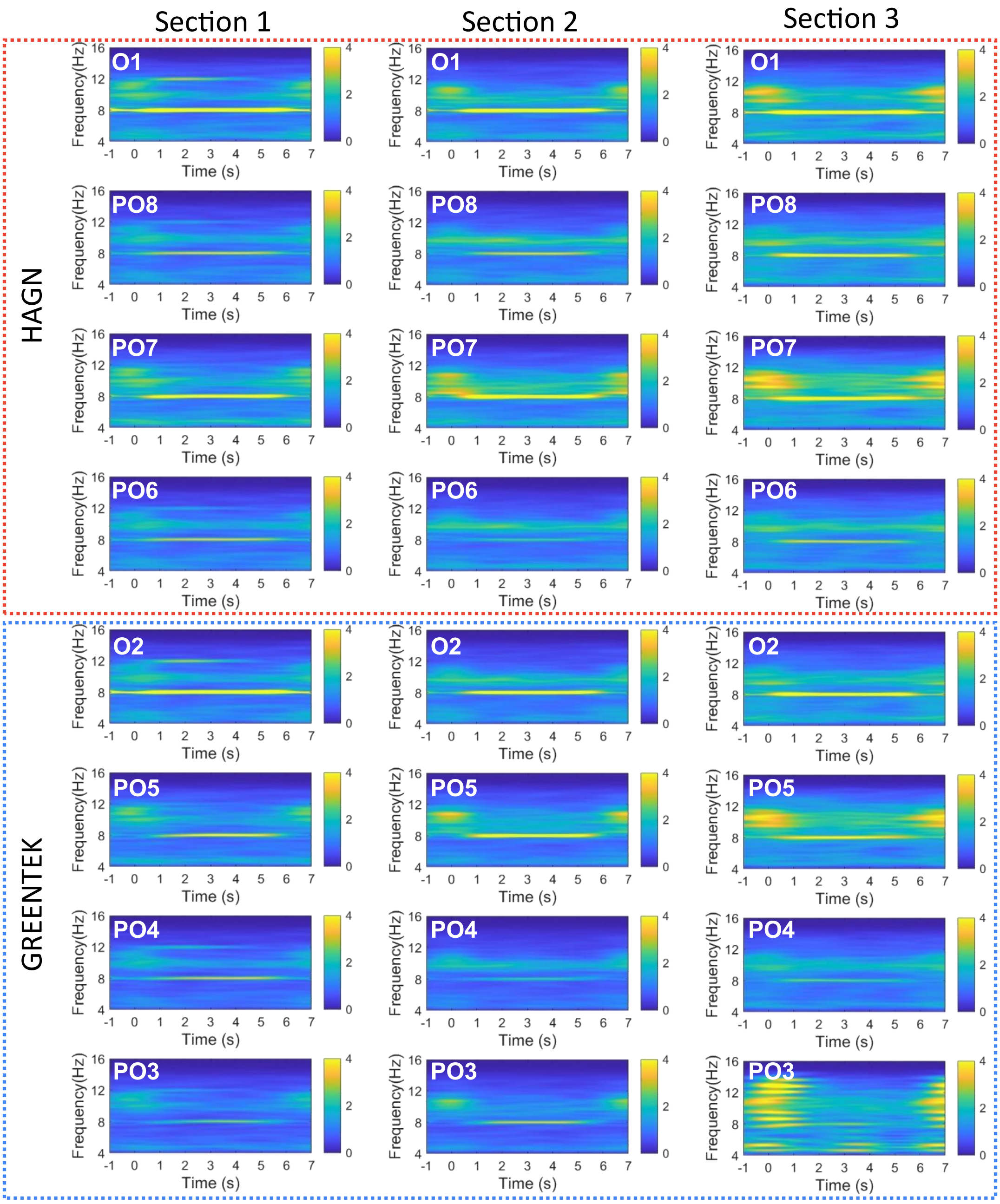

Figure S29. The frequency spectrogram for SSVEP EEG recording of the HAGN hydrogel group and the Greentek gel group in 3 sections.

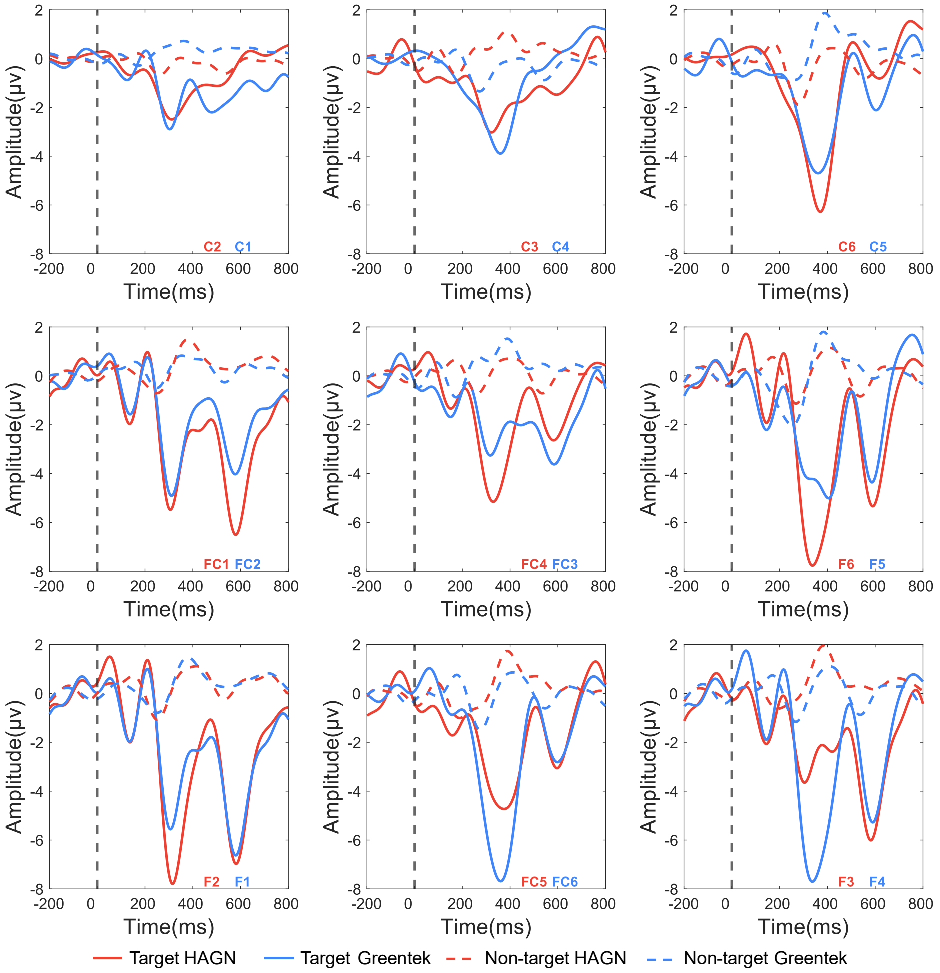

Figure S30. P300 waves of all working channels in section 1.

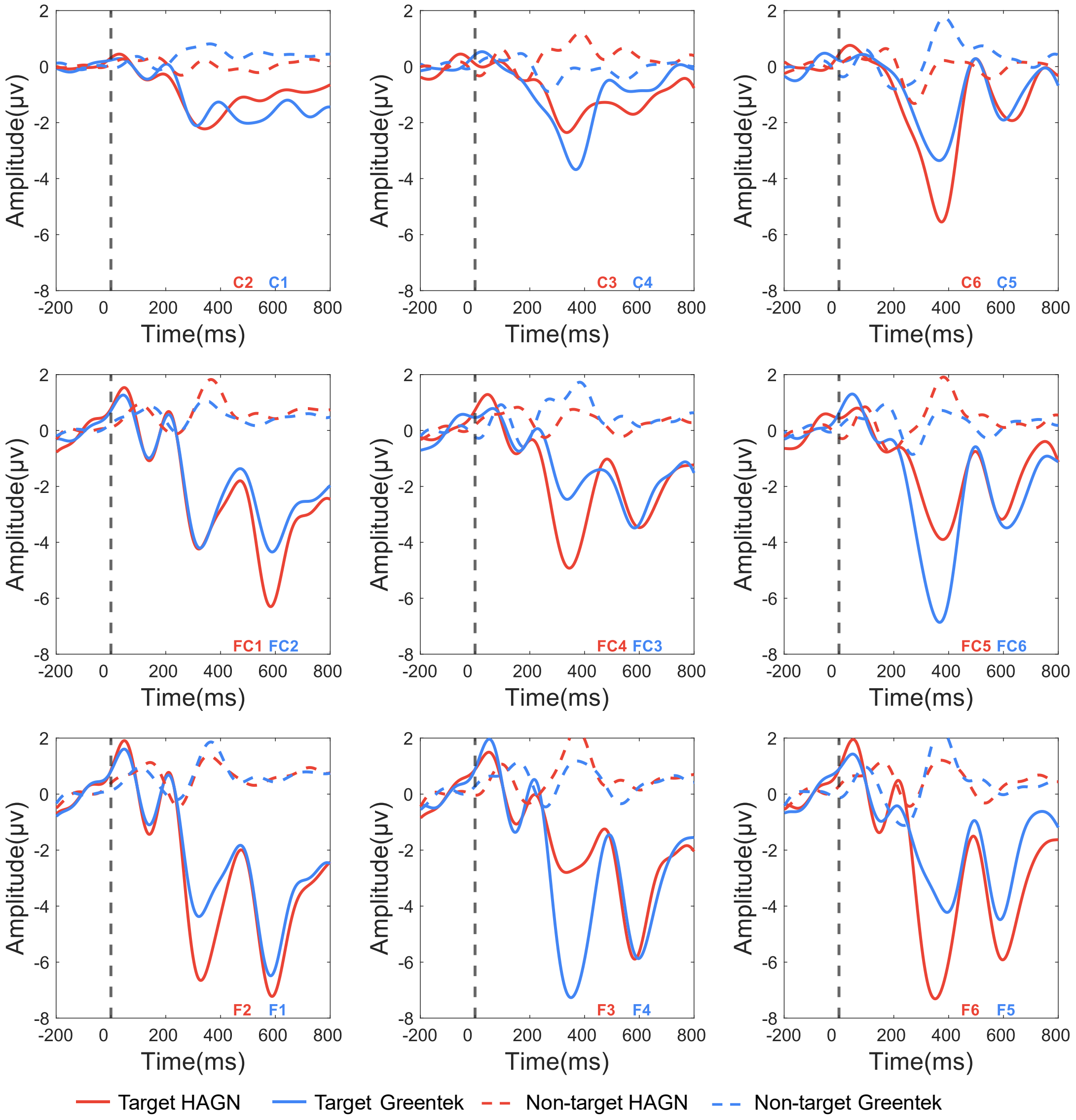

Figure S31. P300 waves of all working channels in section 2.

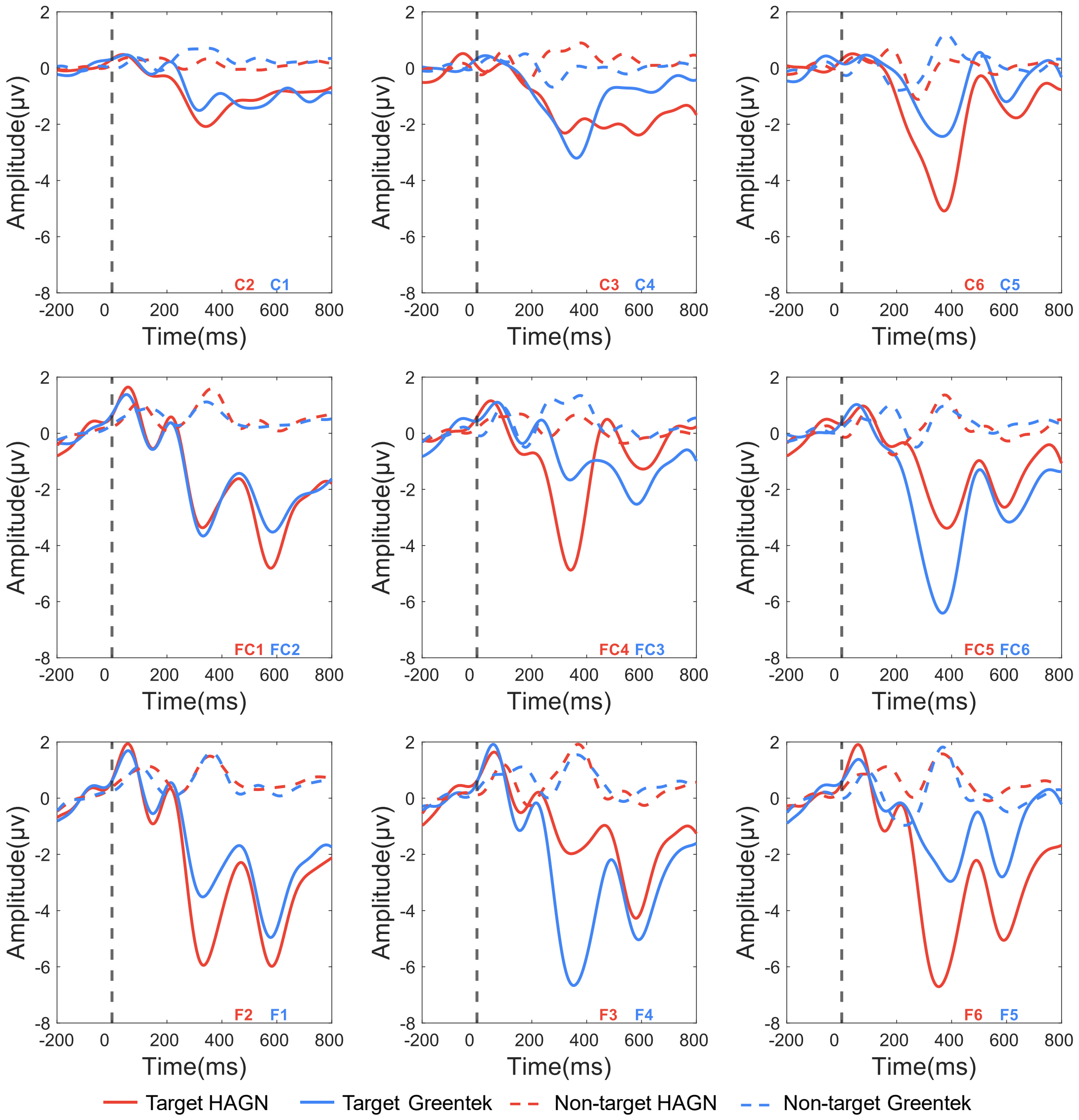

Figure S32. P300 waves of all working channels in section 3.

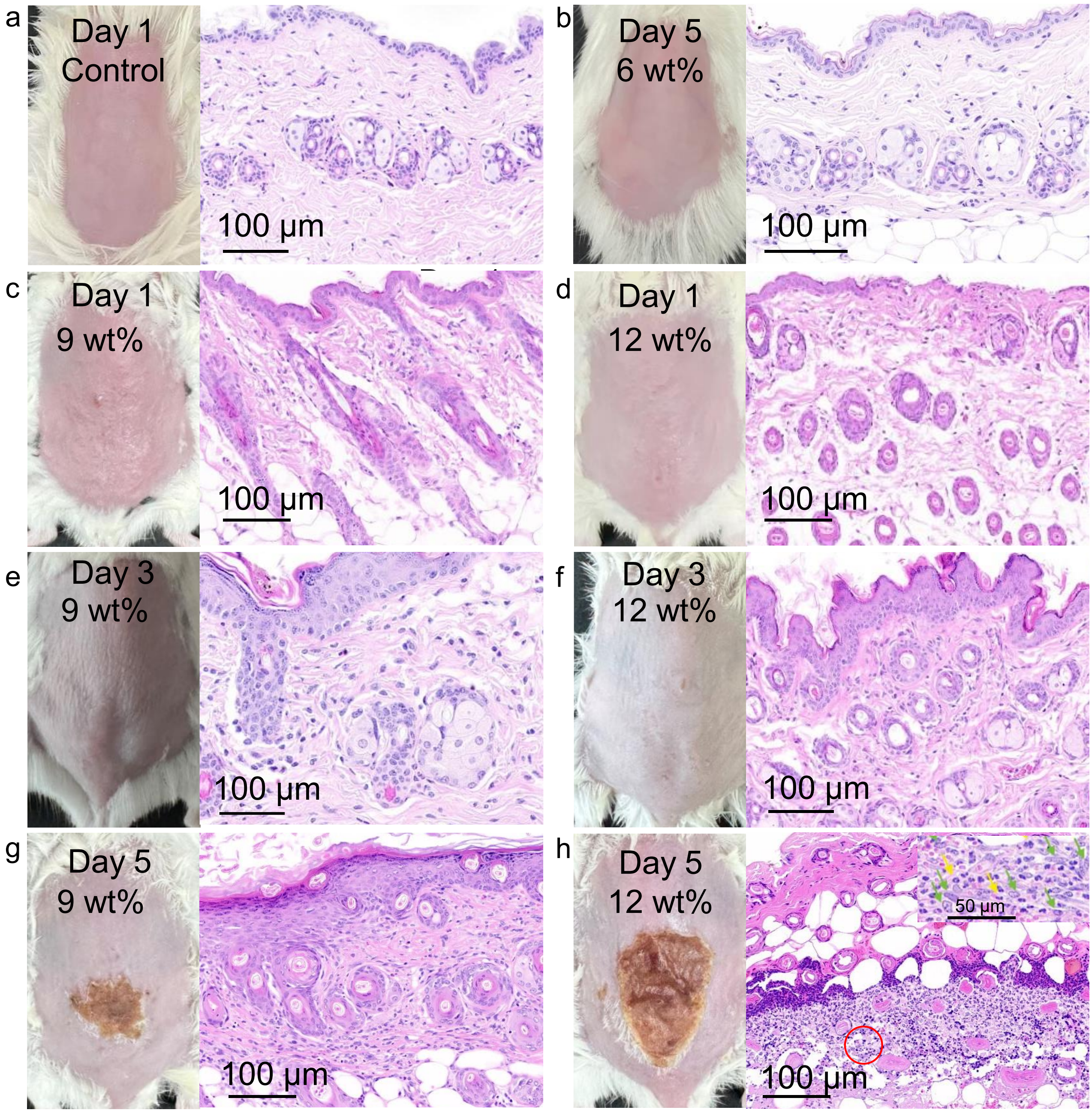

Figure S33. Skin irritation test results of HAGN hydrogels with different salt concentrations, showing mouse bare skin images on the left and H&E stained tissue images on the right for each section**.** (**a**) Control group with no applied gel at day 1; (**b**) 6 wt% NaCl/KCl group at day 5; (**c**) 9 wt% NaCl/KCl group at day 1; (**d**) 12 wt% NaCl/KCl group at day 1; (**e**) 9 wt% NaCl/KCl group at day 3; (**f**) 12 wt% NaCl/KCl group at day 3; (**g**) 9 wt% NaCl/KCl group at day 5; and (**h**) 12 wt% NaCl/KCl group at day 5. Red circles indicate the proliferated neutrophils and macrophages; yellow arrows indicate neutrophils, and green arrows indicate macrophages.

The skin irritation results indicated no adverse reaction for the 6 wt% NaCl/KCl group, while higher concentration groups exhibited skin reactions and histological changes (**Figure S32**). Mice exposed to higher salt concentrations showed redness on the skin by day 1 (**Figure S32 c & d**) and sores and scars by day 5 (**Figure S32 g & h**). Histological analysis revealed eosinophil proliferation in the dermis of these groups (**Figure S32 g & h**). Acute inflammation, evidenced by the presence of neutrophils and macrophages, was observed in the 12 wt% NaCl/KCl group (**Figure S32 h**).

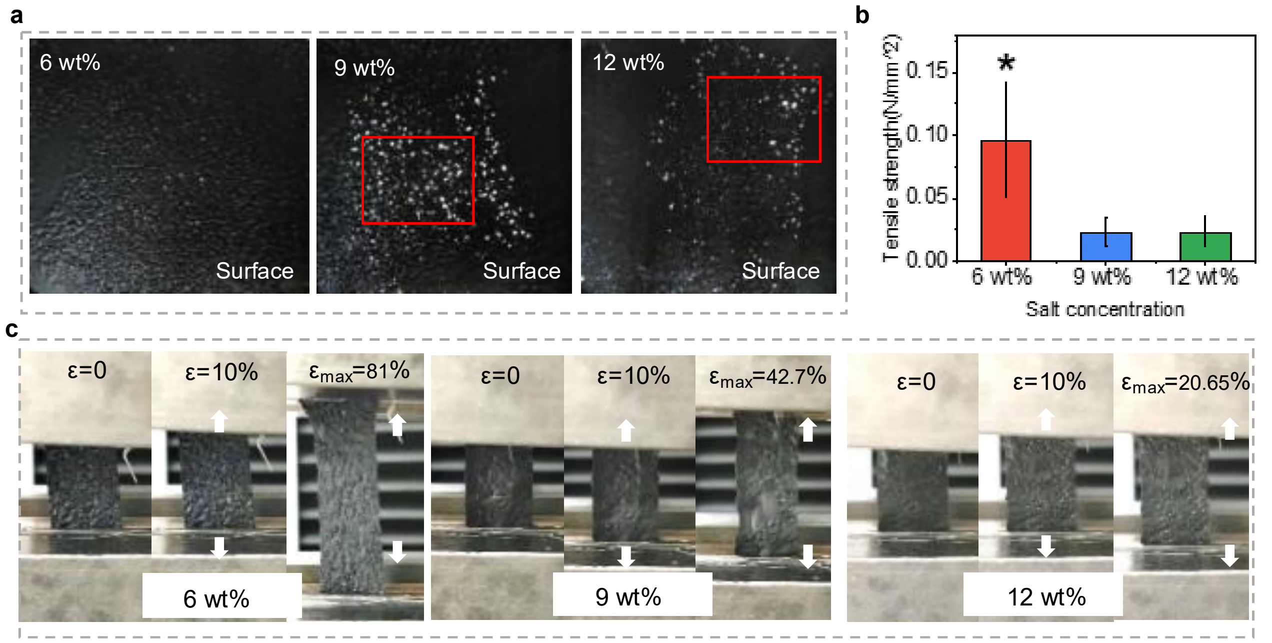

Figure S34. Mechanical properties of HAGN hydrogels with different salt concentrations. (**a**) Surface images of HAGN films with three salt concentrations (6 wt% NaCl/KCl, 9 wt% NaCl/KCl and 12 wt% NaCl/KCl) after 72 hours curing at room temperature; (**b**) Tested tensile strength (Rm) of HAGN films with three salt concentrations; (**c**) The photos of HAGN films under stretching at different degree (initial (ε=0), ε= 10 %; stretched to the maximum force (ε_{max})).

Table

Table S1 Summary of recent hydrogels for EEG electrodes

Ref.	Hydrogel	Working form*	Conductivity	Skin-contact impedance	Duration
[1]	PVA/PVP	Solidified hydrogel	-	3~4 kΩ （1-100 Hz）	-
[2]	PDA/CNT	Solidified hydrogel	8.2 $S \cdot m^{-1}$	-	-
[3]	Fe-SL-g-PAA hydrogel	Solidified hydrogel	7.0 × 10^{-2} $S \cdot m^{-1}$	-	-
[4]	Au/ (PEDOT:PSS))	Solidified hydrogel	Similar than those of commercial electrodes (at 1 kHz)	-	3 days
[5]	(PEDOT) /(PSS),	Solidified hydrogel	-	<0.4 kΩ Wet electrode; ~15 kΩ dry electrode	10 hs
[6]	PEDOT:PSS/PVA conducting hydrogel	Solidified hydrogel	10 S cm^{-1}	2.5–35.1 Ω	-
[7]	AAm/MAAc/EGaIn	Solidified hydrogel	6~10 $S \cdot m^{-1}$	-	-
[8]	PAA/PANI	Solidified hydrogel	5.12 $S \cdot m^{-1}$	-	-
[9]	alginate-based hydrogels	Flowable conductive gels		6 kΩ ~ 25 kΩ (0.5 Hz)	180 mins
[10]	PAAS-MXene	Flowable conductive gels	2.3 $S \cdot m^{-1}$	<50 Ω	-
[11]	FDA/Gelatin/Sodium Chloride	Flowable conductive gels	-	6.95 ± 0.97 kΩ (1 kHz)	1 day
Commercial gels (Greentek)	Commercial electrolytic gels	Flowable conductive gels	S_{max}=2.67 $S \cdot m^{-1}$ S_{min}=0.0078 $S \cdot m^{-1}$	~5 kΩ （wet state） ~19 kΩ (dry state) (1 kHz)	~20 hours
This work	HAGN-50	Flowable conductive gels	S_{max}=4.93 $S \cdot m^{-1}$ S_{min}=1.84 $S \cdot m^{-1}$	~1 kΩ （wet state） ~10 kΩ (dry state) (1 kHz)	72 hours

*Solidified hydrogel electrodes are pre-cut to the appropriate size for attachment to the skin, while flowable conductive gels are injected into the electrode cavity present in most EEG caps.

References

[1] Q. Han, C. Zhang, T. Guo, Y. Tian, W. Song, J. Lei, Q. Li, A. Wang, M. Zhang, S. Bai, X. Yan, *Adv Mater* **2023**, *35* (12), e2209606, https://doi.org/10.1002/adma.202209606.

[2] L. Han, K. Liu, M. Wang, K. Wang, L. Fang, H. Chen, J. Zhou, X. Lu, *Advanced Functional Materials* **2017**, *28* (3), https://doi.org/10.1002/adfm.201704195.

[3] Q. Wang, X. Pan, C. Lin, X. Ma, S. Cao, Y. Ni, *Chemical Engineering Journal* **2020**, *396*, 125341, https://doi.org/https://doi.org/10.1016/j.cej.2020.125341.

[4] P. Leleux, C. Johnson, X. Strakosas, J. Rivnay, T. Herve, R. M. Owens, G. G. Malliaras, *Adv Healthc Mater* **2014**, *3* (9), 1377, https://doi.org/10.1002/adhm.201300614.

[5] H. Xue, D. Wang, M. Jin, H. Gao, X. Wang, L. Xia, D. a. Li, K. Sun, H. Wang, X. Dong, C. Zhang, F. Cong, J. Lin, *Microsystems & Nanoengineering* **2023**, *9* (1), https://doi.org/10.1038/s41378-023-00524-0.

[6] G. Li, K. Huang, J. Deng, M. Guo, M. Cai, Y. Zhang, C. F. Guo, *Advanced Materials* **2022**, *34* (15), 2200261, https://doi.org/https://doi.org/10.1002/adma.202200261.

[7] X. Sui, H. Guo, C. Cai, Q. Li, C. Wen, X. Zhang, X. Wang, J. Yang, L. Zhang, *Chemical Engineering Journal* **2021**, *419*, 129478, https://doi.org/https://doi.org/10.1016/j.cej.2021.129478.

[8] Z. Wang, H. Zhou, J. Lai, B. Yan, H. Liu, X. Jin, A. Ma, G. Zhang, W. Zhao, W. Chen, *Journal of Materials Chemistry C* **2018**, *6* (34), 9200, https://doi.org/10.1039/C8TC02505C.

[9] P. Pedrosa, P. Fiedler, L. Schinaia, B. Vasconcelos, A. C. Martins, M. H. Amaral, S. Comani, J. Haueisen, C. Fonseca, *Sensors and Actuators B: Chemical* **2017**, *247*, 273, https://doi.org/10.1016/j.snb.2017.02.164.

[10] J. Luo, C. Sun, B. Chang, Y. Jing, K. Li, Y. Li, Q. Zhang, H. Wang, C. Hou, *ACS Nano* **2022**, *16* (11), 19373, https://doi.org/10.1021/acsnano.2c08961.

[11] C. Wang, H. Wang, B. Wang, H. Miyata, Y. Wang, M. O. G. Nayeem, J. J. Kim, S. Lee, T. Yokota, H. Onodera, T. Someya, *Sci Adv* **2022**, *8* (20), eabo1396, https://doi.org/10.1126/sciadv.abo1396.

Table S2 The average signal-to-noise ratio (SNR, mean ± std) of SSVEP for the HAGN gel and Greentek gel in sections 1, 2, and 3

section	1		2		3	
type	Greentek	HAGN	Greentek	HAGN	Greentek	HAGN
Mean ±Std	0.83±2.45	0.76±2.29	-0.7±2.29	-1±2.03	-1.53±1.74	-2.13±2.23

No significant differences were observed among the groups in this table.

Table S3 The maximum amplitude (μV) of P300 (mean ± std) recorded by the HAGN gel and Greentek gel in sections 1, 2, and 3

section	1		2		3	
type	GREEN	HAGN	GREEN	HAGN	GREEN	HAGN
Mean ±Std	-1.24±0.46	-1.226±0.23	-0.95±0.48	-0.1±0.3	-0.77±0.78	-0.8±0.67

No significant differences were observed among the groups in this table.

Table S4 Solute concentrations of prepared samples

Sample name	Graphite nanoparticles (GN) % (w/v)	Glycerin % (v/v)	1,2-propanediol % (v/v)	NaCl % (w/v)	KCl % (w/v)	Hyaluronic acid % (w/v)
HAGN-0	0	5	2.5	6	6	5
HAGN-10 (10 mg/ml GN)	1	5	2.5	6	6	5
HAGN-20 (20 mg/ml GN)	2	5	2.5	6	6	5
HAGN-30 (30 mg/ml GN)	3	5	2.5	6	6	5
HAGN-40 (40 mg/ml GN)	4	5	2.5	6	6	5
**HAGN-50 (50 mg/ml GN, 6 wt% NaCl/KCl)**	**5**	**5**	**2.5**	**6**	**6**	**5**
HAGN-60 (60 mg/ml GN)	6	5	2.5	6	6	5
HAGN-100 (100 mg/ml GN)	10	5	2.5	6	6	5
9 wt% NaCl/KCl	5	5	2.5	9	9	5
12 wt% NaCl/KCl	5	5	2.5	12	12	5

The HAGN-50 was selected as the most suitable sample for performing the EEG test.

Table S5 Representative pH values of prepared samples

Sample name	pH value
HAGN-0	6.76±0.01
HAGN-10	6.58±0.01
HAGN-50 (6 wt% NaCl/KCl)	6.61±0.01
9 wt% NaCl/KCl	6.60±0.02
12 wt% NaCl/KCl	6.63±0.01
